# Quasistationarity and extinction for population processes under asymptotic reversibility conditions

**DOI:** 10.1007/s00285-025-02304-y

**Published:** 2025-10-23

**Authors:** Damian Clancy

**Affiliations:** Department of Actuarial Mathematics and Statistics, Maxwell Institute for Mathematical Sciences, Heriot-Watt University, Edinburgh, EH14 4AS UK

**Keywords:** Competition processes, Kolmogorov reversibility criterion, Large deviations, Metastability, Multitype birth and death processes, Stochastic epidemics, 60J28, 92D30, 92D25

## Abstract

We consider stochastic population processes that are almost surely absorbed at the origin within finite time. Our interest is in the quasistationary distribution, ${\varvec{u}}$, and the expected time, $\tau$, from quasistationarity to extinction, both of which we study via WKB approximation. This approach involves solving a Hamilton-Jacobi partial differential equation specific to the model. We provide conditions under which analytical solution of the Hamilton-Jacobi equation is possible, and give the solution. This provides a first approximation to both ${\varvec{u}}$ and $\tau$. We provide further conditions under which a corresponding ‘transport equation’ may be solved, leading to an improved approximation of ${\varvec{u}}$. For multitype birth and death processes, we then consider an alternative approximation for ${\varvec{u}}$ that is valid close to the origin, provide conditions under which the elements of this alternative approximation may be found explicitly, and hence derive an improved approximation for $\tau$. We illustrate our results in a number of applications.

## Introduction

### Background

For many biological population models, including models from ecology (Méléard and Villemonais 2012; Ovaskainen and Meerson 2010), epidemiology (Nåsell 1991, 1999; Andersson and Britton 2000; Nåsell 2011; Clancy 2018b) and immunology (Stirk et al. 2010), eventual extinction (either of the whole population or of some sub-population of interest) is certain, but the population can settle to an apparent equilibrium for a long time before extinction occurs. Two particular objects of interest are then (i) the quasistationary distribution, ${\varvec{u}}$, that the process settles to prior to eventual extinction; and (ii) the time taken for extinction to occur. The time from quasistationarity to extinction is in general an exponentially distributed random variable (van Doorn and Pollett 2013), so that its distribution is fully determined by its expected value $\tau$. Provided the relevant quasistationary distribution is known to exist, it is straightforward to write down equations satisfied by ${\varvec{u}}$ and $\tau$, equations (2) and (3) below, but in general far from straightforward to evaluate their solutions. Approximation methods are therefore of great interest.

We consider a sequence of Markov processes $\{ {\varvec{X}}^{(N)} (t): t \ge 0 \}$ on state space $S^{(N)} \subseteq {{\mathbb {Z}}}_+^k$, indexed by a parameter *N* representing ‘typical’ population size, such that the origin is an absorbing state, the remainder of the state space consists of a single communicating class $C^{(N)}$, and absorption at the origin occurs within finite time with probability 1. This sequence of processes is assumed to be density dependent in the sense of chapter 11 of Ethier and Kurtz (2005); that is, transition rates are of the form

1 $$\begin{aligned} P \left( {\varvec{X}}^{(N)} ( t + \delta t ) = {\varvec{x}}+ {\varvec{l}}\mid {\varvec{X}}^{(N)} (t) = {\varvec{x}}\right)= &  N \beta _{{\varvec{l}}} \left( {{\varvec{x}}\over N} \right) \delta t + o ( \delta t ) \nonumber \\  &  \hspace{5mm} \text{ for } {\varvec{x}}\in S^{(N)},\ {\varvec{l}}\in {{\mathcal {L}}}, \end{aligned}$$

for some functions $\beta _{{\varvec{l}}} ( \cdot )$, where $\mathcal {L}$ is a finite set, independent of *N*, consisting of the possible jumps from each state ${\varvec{x}}\in S^{(N)}$. Under mild technical conditions (theorem 11.2.1 of Ethier and Kurtz 2005), the scaled processes ${\varvec{X}}^{(N)}(t)/N$ may be approximated by the solution of a system of ordinary differential equations, system (11) below. We restrict attention to the situation where this deterministic system has a unique stable equilibrium point ${\varvec{y}}^*$ in the interior of ${{\mathbb {R}}}_+^k$, and the origin is an unstable equilibrium point.

For the processes that we consider, the elements of the quasistationary distribution ${\varvec{u}}^{(N)} = \{ u_{{\varvec{x}}}^{(N)}: {\varvec{x}}\in C^{(N)} \}$ satisfy

2 $$\begin{aligned} \sum _{{\varvec{l}}\in {{\mathcal {L}}}} \left( u_{{\varvec{x}}-{\varvec{l}}}^{(N)} \beta _{{\varvec{l}}} \left( {{\varvec{x}}- {\varvec{l}}\over N} \right) - u_{{\varvec{x}}}^{(N)} \beta _{{\varvec{l}}} \left( {{\varvec{x}}\over N} \right) \right)= &  - (\tau ^{(N)} N)^{-1} u_{{\varvec{x}}}^{(N)} \text{ for } {\varvec{x}}\in C^{(N)}, \end{aligned}$$

and the expected time from quasistationarity to extinction, $\tau ^{(N)}$, is given by

3 $$\begin{aligned} \tau ^{(N)}= &  \left( N \sum _{{\varvec{l}}\in {{\mathcal {L}}}} u_{- {\varvec{l}}}^{(N)} \beta _{{\varvec{l}}} \left( - \, {{\varvec{l}}\over N} \right) \right) ^{-1} \end{aligned}$$

(van Doorn and Pollett 2013, see derivation of result 4 below).

A natural strategy, and one widely used in applications (Nåsell 1999; Lloyd et al. 2007; Andersson and Britton 2000; Ross 2006), is to approximate the quasistationary distribution ${\varvec{u}}^{(N)}$ by a multivariate normal distribution. This gives a reasonably good approximation close to the mode at $N {\varvec{y}}^*$, but the approximation becomes less accurate away from the mode, and, in particular, fails in the tail of the distribution close to the origin—see, for example, figure 3 of Lloyd et al. (2007) and figure 4 of Clancy (2018b). The multivariate normal approximation consequently does not, in general, lead to a good approximation for the expected extinction time $\tau ^{(N)}$.

An alternative approach is to approximate $\tau ^{(N)}$ by the expected hitting time at the origin of an approximating diffusion process, evaluated as the solution of a Kolmogorov backward partial differential equation (Wang et al. 2014; Vadillo 2021). This technique can give a reasonably good approximation for finite *N*, but is known to fail in the large *N* limit (Doering et al. 2005; Clancy and Tjia 2018).

Another approach that has been widely used in applications, e.g. Assaf and Meerson (2010), Ovaskainen and Meerson (2010), Assaf and Meerson (2017), Nieddu et al. (2017), Clancy (2018a), Clancy (2018b), Clancy and Stewart (2024), and the approach that we will take, is based around a WKB (Wentzel, Kramers, Brillouin) approximation. The WKB approach involves seeking a solution ${\varvec{u}}^{(N)}$ to equation (2) such that as $N \rightarrow \infty$, writing ${\varvec{y}}= {\varvec{x}}/ N$, the elements of the quasistationary distribution ${\varvec{u}}^{(N)}$ may be written in the form

4 $$\begin{aligned} u^{(N)}_{{\varvec{x}}}= &  M_N \exp \left( - N V ( {\varvec{y}}) - V_0 ( {\varvec{y}}) + O (1/N) \right) \text{ for } {\varvec{x}}\in C^{(N)}, \end{aligned}$$

for some functions $V ( {\varvec{y}})$, $V_0 ( {\varvec{y}})$ that do not depend upon *N*, and some $M_N$ that does not depend upon ${\varvec{y}}$.

The WKB approach is sometimes referred to as ‘semi-rigorous’; as such, we will avoid the terminology of ‘theorem’ and ‘proof’, and instead refer to our ‘results’ and ‘derivations.’ While not fully rigorous, the WKB approach is well established in the literature for the study of quasistationarity and extinction for population processes, and has been found to give results in (reasonably) good agreement with Monte Carlo simulation in numerous applications, e.g. Assaf and Meerson (2017), Black and McKane (2011), Clancy (2018a), Clancy (2018b), Clancy and Stewart (2024), Nieddu et al. (2017).

By neglecting the second order term $V_0 ( {\varvec{y}})$ in the formula (4) and imposing the constraint that $V ( {\varvec{y}})$ be of quadratic form, we recover the multivariate normal approximation. It is thus apparent that the WKB approximation provides considerably greater flexibility than the multivariate normal approximation. In applications, the WKB approximation is found to perform considerably better than the multivariate normal distribution in approximating the quasistationary distribution—see, for example, figure 4 of Clancy (2018b) and figure 4 of Black and McKane (2011).

It may be shown (Assaf and Meerson 2017, see derivation of result 4 below) that the function $V ( {\varvec{y}})$ in the formula (4) satisfies the Hamilton-Jacobi partial differential equation

5 $$\begin{aligned} \sum _{{\varvec{l}}\in {{\mathcal {L}}}} \beta _{{\varvec{l}}} ( {\varvec{y}})\left( \exp \left( {\varvec{l}}^T {\partial V \over \partial {\varvec{y}}} \right) - 1 \right)= &  0, \end{aligned}$$

while $V_0 ( {\varvec{y}})$ satisfies the ‘transport equation’

6 $$\begin{aligned} \sum _{{\varvec{l}}\in {{\mathcal {L}}}} \exp \left( {\varvec{l}}^T {\partial V \over \partial {\varvec{y}}} \right) {\varvec{l}}^T \left( \left( {\partial V_0 \over \partial {\varvec{y}}} - {1 \over 2} {\partial ^2 V \over \partial {\varvec{y}}^2} {\varvec{l}}\right) \beta _{{\varvec{l}}} ( {\varvec{y}}) - {\partial \beta _{{\varvec{l}}} \over \partial {\varvec{y}}} \right)= &  0. \end{aligned}$$

For a variety of 1-dimensional systems, it is possible to solve equations (5), (6) analytically, giving explicit formulae for $V ( {\varvec{y}})$, $V_0 ( {\varvec{y}})$ and $M_N$ (Assaf and Meerson 2010, 2017). The approximation (4) fails in the tail of the distribution close to extinction, since $V_0 ( {\varvec{y}})$ diverges at the origin. However, an alternative approximation to ${\varvec{u}}^{(N)}$ may be found by linearising transition rates close to the origin, giving an un-normalised approximation valid in the relevant tail of the distribution, and then normalising by matching with the WKB approximation. This leads to a relationship of the form

7 $$\begin{aligned} \tau ^{(N)}\sim &  {K \over \sqrt{N}} \exp ( NA ), \end{aligned}$$

where we use $\sim$ to denote that the ratio of the two sides converges to 1 as $N \rightarrow \infty$, and *A*, *K* are constants that can be found in terms of the parameters of the process.

For models in $k>1$ dimensions, it is usually only possible to find the leading order term $V ( {\varvec{y}})$ by solving the Hamilton-Jacobi equation (5) numerically. This leads to results of the cruder form

8 $$\begin{aligned} \lim _{N \rightarrow \infty } \frac{\ln \tau ^{(N)}}{N}= &  A, \end{aligned}$$

where the leading order constant *A* must be evaluated via numerical solution of a system of ordinary differential equations in 2*k* dimensions (Assaf and Meerson 2017; Nieddu et al. 2017; Bauver et al. 2016; Dykman et al. 1994; Kamenev and Meerson 2008; Clancy and Stewart 2025). An exception is provided by Clancy (2018b), where a relation of the form (7) and explicit formulae for *A*, *K* are obtained for a *k* dimensional susceptible-infectious-susceptible (SIS) infection model in a heterogeneous population, example 1 below. Solution is based upon the observation that for the model of Clancy (2018b), the *k* dimensional Hamilton-Jacobi equation (5) for $V ( {\varvec{y}})$ can be separated into *k* components, each of which can be straightforwardly solved as an ordinary differential equation, and then the transport equation (6) can similarly be solved for $V_0 ( {\varvec{y}})$.

A rather different approach was taken in Ball and Clancy (2023) to obtain a relationship of the form (7) together with explicit formulae for *A*,  *K* for a class of multitype birth and death processes, example 3 below. A modified version of the process $X^{(N)} (t)$ is considered, re-started in a particular way each time the process hits the origin. The stationary distribution of this restarted process is found, and used to study the time to extinction of the original process.

### Asymptotic reversibility and analytical solutions

In the current paper, we characterise the class of processes such that the Hamilton-Jacobi equation (5) and the transport equation (6) can be analytically solved (in any number of dimensions) via separation into component ordinary differential equations. We then go on to set out conditions under which a relationship of the form (7) may be obtained. Conditions (24) and (25) below characterise the processes for which an analytical solution to the Hamilton-Jacobi equation (5) may be obtained in this way. When combined with our assumptions 1–8 below, we obtain a relationship of the form (8) with an analytical expression for the constant *A*, result 4 below. The further conditions (29) and (30), in addition to conditions (24) and (25), then characterise the processes for which an analytical solution to the transport equation (6) may be obtained. From this we obtain an analytical approximation to the main body of the quasistationary distribution ${\varvec{u}}^{(N)}$, result 5. Restricting to multitype birth and death processes, we set out in result 3 conditions under which an improved approximation to ${\varvec{u}}^{(N)}$ close to the extinction point at the origin may be obtained, and hence a relationship of the form (7) with explicit expressions for the constants *A*, *K*. The conditions of result 3, our most restrictive conditions, remain less restrictive than those of Ball and Clancy (2023), and indeed we are able to treat the model of Ball and Clancy (2023) as a special case, example 3 below. The results of Ball and Clancy (2023) are the most general previously available results that we are aware of in this vein.

Our approach relies throughout upon asymptotic (weaker) versions of Kolmogorov’s criterion for reversibility (Kelly 2011). Kolmogorov’s criterion states that for an irreducible, positive recurrent Markov process with state space *S*, if the transition rates $\{ q_{{\varvec{x}},{\varvec{x}}^\prime }: {\varvec{x}}, {\varvec{x}}^\prime \in S \}$ satisfy

9 $$\begin{aligned} q_{{\varvec{x}}_1, {\varvec{x}}_2} q_{{\varvec{x}}_2, {\varvec{x}}_3} \cdots q_{{\varvec{x}}_{n-1}, {\varvec{x}}_n} q_{{\varvec{x}}_n, {\varvec{x}}_1}= &  q_{{\varvec{x}}_n, {\varvec{x}}_{n-1}} q_{{\varvec{x}}_{n-1}, {\varvec{x}}_{n-2}} \cdots q_{{\varvec{x}}_2, {\varvec{x}}_1} q_{{\varvec{x}}_1, {\varvec{x}}_n} \end{aligned}$$

for every finite sequence of states ${\varvec{x}}_1, {\varvec{x}}_2, \ldots , {\varvec{x}}_n \in S$, then the stationary distribution $\varvec{\pi }$ of the process satisfies the detailed balance equations

10 $$\begin{aligned} \varvec{\pi }_{{\varvec{x}}} q_{{\varvec{x}}, {\varvec{x}}^\prime }= &  \varvec{\pi }_{{\varvec{x}}^\prime } q_{{\varvec{x}}^\prime ,{\varvec{x}}} \text{ for } {\varvec{x}}, {\varvec{x}}^\prime \in S. \end{aligned}$$

The processes ${\varvec{X}}^{(N)} (t)$ that we consider are not irreducible, having an absorbing state at the origin, and a unique stationary distribution that assigns probability 1 to the state ${\varvec{x}}= \textbf{0}$. However, we shall show that under various asymptotic versions of the Kolmogorov criterion (9), asymptotic versions of detailed balance hold, allowing us to find explicitly the functions $V ( {\varvec{y}})$, $V_0 ( {\varvec{y}})$ and constant $M_N$ in the asymptotic approximation (4), as well as an alternative approximation to ${\varvec{u}}^{(N)}$ valid in the tail of the distribution close to extinction. The relevant asymptotic Kolmogorov criteria are given as equations (24), (25), (29), (30), (21) below. We discuss the interpretation of these various criteria in remark 5 following the statements of our results in Sect. 3, see also remark 6 following our derivations in Sect. 6.

Our main conclusions are as follows: under assumptions 1–8 below, then (i) the function $V ( {\varvec{y}})$, and hence a relationship of the form (8), may be found explicitly when conditions (24) and (25) are satisfied; (ii) the function $V_0 ( {\varvec{y}})$, and hence an approximation to ${\varvec{u}}^{(N)}$ of the form (4), may be obtained when, in addition, conditions (29) and (30) are satisfied; (iii) a relationship of the form (7) may be obtained when, in addition, the process of interest is a multitype birth and death process and conditions (13) and (21) are satisfied.

Our formula (23) giving the asymptotic form of the time to extinction from quasistationarity is analogous to the Eyring-Kramers formula (Berglund 2013) characterising the expected transition time between locally stable equilibrium points for a reversible diffusion process. For processes on a discrete state space, the recent paper Jia et al. 2021 studies the exit time and exit distribution from a basin of attraction of a locally stable equilibrium point for chemical reaction network models, so that transition rate functions are restricted to be of the form shown on p889 of Jia et al. (2021). Theorem 4 of Jia et al. (2021) parallels, in this somewhat different context, our result 4 below.

Following the statement of our results in Sect. 3, in Sect. 4 we exhibit examples of biological population models satisfying the relevant asymptotic Kolmogorov criteria, and for which we can therefore provide explicit results. There are, of course, many other population processes that do not satisfy these criteria. It is of particular interest to understand the conditions under which exact solution of equation (5) is possible because numerical solution of the Hamilton-Jacobi equation (5) otherwise requires the solution of a high-dimensional system of ordinary differential equations (the characteristic equations) subject to boundary conditions at times $t = - \infty$ and $t = + \infty$, see, for example, Clancy and Stewart (2025). The relevant characteristic curve is maximally sensitive to perturbations, so that numerical solution is usually only feasible in low dimensions (Assaf and Meerson 2017; Bauver et al. 2016; Black and McKane 2011; Nieddu et al. 2017). Numerical solution techniques generally require an initial guess of the solution curve, and only work well provided the initial guess is close to the true solution. When a particular population process of interest does not satisfy our conditions (24), (25), but is closely related to a process that does, then one option is to use the explicit solution $V ( {\varvec{y}})$ for the process that does satisfy conditions (24), (25) as our initial guess when numerically solving to find the characteristic curve for the process of interest. An example of this approach appears in Clancy and Stewart (2025).

The value of our results is thus threefold: (i) for processes that satisfy our various asymptotic Kolmogorov criteria, we obtain explicit analytical results; (ii) for processes that do not satisfy our criteria, our results nevertheless open up a possible new route to obtaining numerical results; (iii) our asymptotic Kolmogorov criteria give insight into the structure of population processes that are amenable to a relatively straightforward analysis—in particular, see remark 5 and remark 6 below.

### Outline of the paper

The remainder of the paper is structured as follows. In Sect. 2 we set out our model assumptions, before giving the statements of our results in Sect. 3. Some particular applications are given in Sect. 4, and numerical results for a specific simple model in Sect. 5. Derivations of our results are given in Sect. 6.

**Notation**: for any set $E \subset {{\mathbb {R}}}^k$, we denote by $E^{\circ }$, $\partial E$ the interior and boundary, respectively, of the set *E*. We denote by $d( \cdot , \cdot )$ the Euclidean distance between two points in ${{\mathbb {R}}}^k$ or between a point and a set in ${{\mathbb {R}}}^k$, so that in particular $d ( {\varvec{y}}, \partial E )$ represents the distance from the point ${\varvec{y}}$ to the boundary of the set *E*. For $i=1,2,\ldots ,k$ we denote by ${\varvec{e}}_i$ the unit vector with *i*th element equal to 1.

## Model assumptions

Consider a sequence of Markov processes $\left\{ {\varvec{X}}^{(N)} (t): t \ge 0 \right\}$ on state space $S^{(N)} \subseteq {{\mathbb {Z}}}_+^k$, indexed by a parameter *N*. We adopt the following assumptions throughout.

1. The state space $S^{(N)}$ is either the whole of ${{\mathbb {Z}}}_+^k$ for all *N*, or the finite set $\left\{ {\varvec{x}}= \left( x_ 1, x_2, \ldots , x_k \right) : 0 \le x_i \le N_i \text{ for } i=1,2,\ldots ,k \right\}$ for some ${\varvec{N}}= ( N_1, N_2, \ldots , N_k )$ with $N_1 + N_2 + \cdots + N_k = N$. In the case of finite state space, writing $f_i = N_i / N$ for $i = 1,2, \ldots ,k$, we assume for simplicity that $f_1, f_2, \ldots , f_k$ do not vary with *N* (so when we write, for instance, $N \rightarrow \infty$, it is implicit that *N* increases through a subsequence of integers such that $N f_i$ is always integer-valued for all *i*), and that $f_1, f_2, \ldots , f_k > 0$.
2. The sequence of processes is density dependent in the sense of chapter 11 of Ethier and Kurtz (2005). That is, writing ${\tilde{S}} = {{\mathbb {R}}}_+^k$ in the case that $S^{(N)} = {{\mathbb {Z}}}_+^k$ for all *N*, and ${\tilde{S}} = [ 0, f_1 ] \times [ 0, f_2 ] \times \cdots \times [ 0, f_k ]$ in the case of finite state space, then for some finite set ${{\mathcal {L}}}$, independent of *N*, consisting of the possible jumps from each state ${\varvec{x}}\in S^{(N)}$, and for some functions $\beta _{{\varvec{l}}}: {\tilde{S}} \rightarrow {{\mathbb {R}}}_+$, transition rates are of the form (1).
3. The set ${{\mathcal {L}}}$ spans ${{\mathbb {R}}}^k$, and for every ${\varvec{l}}\in {{\mathcal {L}}}$ we also have $- {\varvec{l}}\in {{\mathcal {L}}}$.
4. For every ${\varvec{l}}\in {{\mathcal {L}}}$ we have $\beta _{{\varvec{l}}} ( \textbf{0} ) = 0$.
5. For all ${\varvec{y}}\in {\tilde{S}} \setminus \{ \textbf{0} \}$, ${\varvec{l}}\in {{\mathcal {L}}}$, we have $\beta _{{\varvec{l}}} ( {\varvec{y}}) > 0$ unless $y_i = 0$ and $l_i < 0$ for some *i* or (in the case of finite state space) $y_i = f_i$ and $l_i > 0$ for some *i*, in which case $\beta _{{\varvec{l}}} ( {\varvec{y}}) = 0$.
6. The functions $\beta _{{\varvec{l}}} ( \cdot )$ are continuous on ${\tilde{S}}$ and twice differentiable on ${\tilde{S}}^\circ$, with finite first derivatives (defined in a one-sided sense) at the origin.
7. The ordinary differential equation system
  11 $$\begin{aligned} {d {\varvec{y}}\over dt}= &  \sum _{{\varvec{l}}\in {{\mathcal {L}}}} {\varvec{l}}\beta _{{\varvec{l}}} ( {\varvec{y}}) \end{aligned}$$
  has two equilibrium points in ${\tilde{S}}$: a locally unstable equilibrium point at ${\varvec{y}}= \textbf{0}$ and a locally stable equilibrium point ${\varvec{y}}^* \in {\tilde{S}}^\circ$, with all eigenvalues of the Jacobian of (11) at ${\varvec{y}}^*$ having strictly negative real part.
8. For each *N* there exists a unique (proper) limiting conditional distribution ${\varvec{u}}^{(N)} = \{ u^{(N)}_{{\varvec{x}}}: {\varvec{x}}\in C^{(N)} \}$ such that for every initial state ${\varvec{x}}_0 \in C^{(N)}$,
  $$\begin{aligned} u_{{\varvec{x}}}^{(N)}= &  \lim _{t \rightarrow \infty } \Pr \left( {\varvec{X}}^{(N)}(t) = {\varvec{x}}\left| {\varvec{X}}^{(N)} ( 0 ) = {\varvec{x}}_0, \, {\varvec{X}}^{(N)} (t) \in C^{(N)} \right. \right) \text{ for } {\varvec{x}}\in C^{(N)}. \end{aligned}$$

Assumption 4 ensures that the state ${\varvec{x}}= \textbf{0}$ is absorbing, while assumption 5 ensures that $C^{(N)} = S^{(N)} \setminus \{ \textbf{0} \}$ forms a single communicating class. We expect versions of our results to hold if assumption 5 is replaced by the weaker assumption that for all *N*, $C^{(N)}$ forms a single communicating class, but the derivations would then become more involved, so we retain assumption 5 for simplicity. Although we will not make use of this result, we observe that under mild conditions, by Theorem 11.2.1 of Ethier and Kurtz (2005), the scaled processes ${\varvec{X}}^{(N)} (t) / N$ converge almost surely over finite time intervals, as $N \rightarrow \infty$, to the solution ${\varvec{y}}(t)$ of the system (11). In the case of a finite state space, assumption 8 is automatically satisfied (Darroch and Seneta 1967). In the case of an infinite state space, checking assumption 8 can be far from trivial; we refer the reader to van Doorn and Pollett (2013), Chazottes et al. (2019), Champagnat and Villemonais (2023) for relevant results and discussion. By theorem 13 and corollaries 7 and 9 of van Doorn and Pollett (2013), it follows from assumption 8 that ${\varvec{u}}^{(N)}$ is a minimal quasistationary distribution for the process ${\varvec{X}}^{(N)} (t)$, that ${\varvec{X}}^{(N)} (t)$ is almost surely absorbed at $\textbf{0}$ within finite time, and that for the process initiated from quasistationarity, so ${\varvec{X}}^{(N)} (0) \sim {\varvec{u}}^{(N)}$, the time until absorption at the origin is exponentially distributed.

## Results

Recall that ${\varvec{u}}^{(N)}$ denotes the quasistationary distribution of the process, and $\tau ^{(N)}$ the expected time to extinction given that the state of the process at $t=0$ is distributed according to ${\varvec{u}}^{(N)}$. Since our results simplify significantly in the case of multitype birth and death processes, we present this case first, before moving on in section 3.2 to more general population processes.

### Multitype birth and death processes

In the case ${{\mathcal {L}}} = \{ {\varvec{e}}_i, - {\varvec{e}}_i: i=1,2,\ldots ,k \}$, we have the following results.

#### Result 1

For $i, j \in \{ 1,2,\ldots ,k \}$, denote by $b_{ij}$, $d_i$ the constants

12 $$\begin{aligned} b_{ij} = \left. \frac{\partial \beta _{{\varvec{e}}_i}}{\partial y_j} \right| _{{\varvec{y}}= \textbf{0}}, &  d_i = \left. \frac{\partial \beta _{-{\varvec{e}}_i}}{\partial y_i} \right| _{{\varvec{y}}= \textbf{0}}, \end{aligned}$$

and suppose that

13 $$\begin{aligned} d_i> 0 \text{ for } i=1,2,\ldots ,k, \text{ and } \text{ for } \text{ each } i, \text{ there } \text{ exists } \text{ some } j \text{ with } b_{ij} > 0. \end{aligned}$$

Define the vector field ${\varvec{h}}( {\varvec{y}}) = \left( h_1 ( {\varvec{y}}), h_2 ( {\varvec{y}}), \ldots , h_k ( {\varvec{y}}) \right)$ to have components

14 $$\begin{aligned} h_i ( {\varvec{y}})= &  \ln \left( \frac{\beta _{-{\varvec{e}}_i} ({\varvec{y}})}{\beta _{{\varvec{e}}_i} ({\varvec{y}})} \right) \text{ for } i=1,2,\ldots ,k. \end{aligned}$$

If for all $i,j \in \{ 1,2,\ldots ,k \}$ and ${\varvec{y}}\in {\tilde{S}}^\circ$ we have

15 $$\begin{aligned} {\partial h_i \over \partial y_j}= &  {\partial h_j \over \partial y_i}, \end{aligned}$$

then

16 $$\begin{aligned} \frac{\ln \tau ^{(N)}}{N}\rightarrow &  \int _{\Gamma _0} {\varvec{h}}( {\varvec{y}}^\prime ) \cdot d{\varvec{y}}^\prime \text{ as } N \rightarrow \infty , \end{aligned}$$

where $\Gamma _0$ is any path from ${\varvec{y}}^*$, the locally stable equilibrium point of the system (11), to $\textbf{0}$ that lies entirely within ${\tilde{S}}$, and the integral is independent of the particular path $\Gamma _0$.

#### Remark 1

The multitype birth and death process ${\varvec{X}}^{(N)} (t)$ may be approximated close to the origin, for large *N*, by a *linear* multitype birth and death process in which each type *j* individual gives birth to type *i* individuals at rate $b_{ij}$, and individuals of type *i* die at per capita rate $d_i$, where $b_{ij}$, $d_i$ are given by equations (12) (note that, by assumption (5), $\left. \partial \beta _{-{\varvec{e}}_i} / \partial y_j \right| _{{\varvec{y}}= \textbf{0}} = 0$ for $i \ne j$). The condition within assumption 7 that the equilibrium point of system (11) at ${\varvec{y}}= \textbf{0}$ is locally unstable corresponds to the approximating linear process being supercritical (Diekmann et al. 2010, Theorem A.1). In the derivation of result 1, conditions (13) ensure that the integral on the right hand side of the relationship (16) converges. We make greater use of the approximating linear process in the derivation of result 3 in Sect. 6.5.

#### Result 2

With functions $h_i ( {\varvec{y}})$ given by equation (14), denote by $\Sigma$ the matrix with entries

17 $$\begin{aligned} \sigma _{ij}= &  \left. \frac{\partial h_i}{\partial y_j} \right| _{{\varvec{y}}= {\varvec{y}}^*} \text{ for } i,j = 1,2,\ldots ,k. \end{aligned}$$

Define the vector field ${\varvec{h}}^0 ( {\varvec{y}}) = \left( h^0_1 ( {\varvec{y}}), h^0_2 ( {\varvec{y}}) \ldots , h^0_k ( {\varvec{y}}) \right)$ to have components

18 $$\begin{aligned} h^0_i ( {\varvec{y}})= &  {1 \over 2} {\partial \over \partial y_i} \ln \left( \beta _{{\varvec{e}}_i} ( {\varvec{y}}) \beta _{-{\varvec{e}}_i} ( {\varvec{y}}) \right) \text{ for } i=1,2,\ldots ,k. \end{aligned}$$

If, in addition to condition (15), we have that for all $i,j \in \{ 1,2,\ldots ,k \}$ and ${\varvec{y}}\in {\tilde{S}}^\circ$,

19 $$\begin{aligned} {\partial h^0_i \over \partial y_j}= &  {\partial h^0_j \over \partial y_i}, \end{aligned}$$

then writing ${\varvec{y}}= {\varvec{x}}/ N$, the body of the quasistationary distribution may be approximated as

20 $$\begin{aligned} u_{{\varvec{x}}}^{(N)}= &  \sqrt{\frac{\text{ det } ( \Sigma )}{(2 \pi N)^k} \prod _{i=1}^k \frac{\beta _{{\varvec{e}}_i} \left( y_1, \ldots , y_{i-1}, y_i^*, \ldots , y_k^* \right) \beta _{-{\varvec{e}}_i} \left( y_1, \ldots , y_{i-1}, y_i^*, \ldots , y_k^* \right) }{\beta _{{\varvec{e}}_i} \left( y_1, \ldots , y_i, y_{i+1}^*, \ldots , y_k^* \right) \beta _{-{\varvec{e}}_i} \left( y_1, \ldots , y_i, y_{i+1}^*, \ldots , y_k^* \right) }} \nonumber \\  &  {} \times \exp \left( - N \int _{\Gamma } {\varvec{h}}( {\varvec{y}}^\prime ) \cdot d{\varvec{y}}^\prime + O (1/N) \right) \end{aligned}$$

for ${\varvec{y}}\in {\tilde{S}}^\circ$ with $d ( {\varvec{y}}, \partial {\tilde{S}} ) \ne o(1)$, where $\Gamma$ is any path from ${\varvec{y}}^*$ to ${\varvec{y}}$ that lies entirely within ${\tilde{S}}$, the integral being independent of the particular path $\Gamma$.

#### Remark 2

It is standard to approximate the quasistationary distribution ${\varvec{u}}^{(N)}$ by a multivariate normal distribution with mean $N {\varvec{y}}^*$ and variance matrix $N \Sigma ^{-1}$, where the matrix $\Sigma$ has elements $\sigma _{ij}$ given by equation (17) (and for more general processes, the matrix $\Sigma$ is given by equation (28) below). Formula (20) (and formula (32) for more general processes) gives a significantly improved approximation, particularly away from the mode of the distribution—see Fig. 2 below.

#### Remark 3

Conditions (15), (19) simply state that, in the language of vector calculus, the vector fields ${\varvec{h}}( {\varvec{y}})$, ${\varvec{h}}^{(0)} ( {\varvec{y}})$, respectively, are irrotational. The equivalent conditions for more general processes are given as conditions (25), (30) below. For interpretation of these conditions, see remarks 5 and 6.

#### Result 3

If, in addition to conditions (13), (15) and (19), we have, recalling the definitions (12), that for all $i,j \in \{ 1,2,\ldots ,k \}$,

21 $$\begin{aligned} b_{ij}= &  b_{ii}, \end{aligned}$$

then denoting by *D* the unique positive solution of

22 $$\begin{aligned} \sum _{i=1}^k \frac{b_{ii}}{D + d_i}= &  1, \end{aligned}$$

we have

23 $$\begin{aligned} \tau ^{(N)}\sim &  \frac{1}{D} \sqrt{\frac{2 \pi }{N \text{ det } ( \Sigma )} {b_{kk} \prod _{i=1}^k \left. \frac{\partial \beta _{-{\varvec{e}}_i}}{\partial y_i} \right| _{{\varvec{y}}= (0, \ldots , 0, y^*_{i+1}, \ldots , y^*_k )} \prod _{i=1}^{k-1} \beta _{{\varvec{e}}_i} \left( 0, \ldots , 0, y_{i+1}^*, \ldots , y_k^* \right) \over \prod _{i=1}^k \beta _{{\varvec{e}}_i} \left( 0, \ldots , 0, y_i^*, \ldots , y_k^* \right) \beta _{-{\varvec{e}}_i} \left( 0, \ldots , 0, y_i^*, \ldots , y_k^* \right) }} \nonumber \\ &  {} \times \exp \left( N \int _{\Gamma _0} {\varvec{h}}( {\varvec{y}}^\prime ) \cdot d{\varvec{y}}^\prime \right) \text{ as } N \rightarrow \infty , \end{aligned}$$

where $\Gamma _0$ is any path from ${\varvec{y}}^*$ to $\textbf{0}$ that lies entirely within ${\tilde{S}}$, the integral being independent of the particular path $\Gamma _0$.

#### Remark 4

It is not immediately apparent that the formula (23) is invariant under re-labelling of the coordinate axes. That this is indeed the case under the conditions of result 3 follows from the derivation of the result (section 6.5), and becomes clear in specific applications, see section 4 below.

### General population processes

Moving on to more general population processes, result 4 below generalises result 1 above, while result 5 generalises result 2.

#### Result 4

Suppose that for $a_1, a_2, \ldots , a_n \in {{\mathbb {Z}}}$ and ${\varvec{l}}_1, {\varvec{l}}_2, \ldots , {\varvec{l}}_n \in {{\mathcal {L}}}$ we have

24 $$\begin{aligned} \sum _{i=1}^n a_i {\varvec{l}}_i = \textbf{0}\Rightarrow &  \prod _{i=1}^n \left( \frac{\beta _{-{\varvec{l}}_i} ( {\varvec{y}})}{\beta _{{\varvec{l}}_i} ( {\varvec{y}})} \right) ^{a_i} = 1 \text{ for } \text{ all } {\varvec{y}}\in {\tilde{S}}^\circ , \end{aligned}$$

and that

25 $$\begin{aligned} {\varvec{l}}_1^T \frac{\partial }{\partial {\varvec{y}}} \ln \left( \frac{\beta _{-{\varvec{l}}_2} ( {\varvec{y}})}{\beta _{{\varvec{l}}_2} ( {\varvec{y}})} \right)= &  {\varvec{l}}_2^T \frac{\partial }{\partial {\varvec{y}}} \ln \left( \frac{\beta _{-{\varvec{l}}_1} ( {\varvec{y}})}{\beta _{{\varvec{l}}_1} ( {\varvec{y}})} \right) \text{ for } \text{ every } {\varvec{l}}_1, {\varvec{l}}_2 \in {{\mathcal {L}}}. \end{aligned}$$

Then the system of equations

26 $$\begin{aligned} {\varvec{l}}^T \varvec{\theta }( {\varvec{y}})= &  \ln \left( \frac{\beta _{-{\varvec{l}}} ( {\varvec{y}})}{\beta _{{\varvec{l}}} ( {\varvec{y}})} \right) \text{ for } \text{ all } {\varvec{l}}\in {{\mathcal {L}}} \end{aligned}$$

has a unique solution $\varvec{\theta }( {\varvec{y}})$ for each ${\varvec{y}}\in \tilde{S}^\circ$, and provided the integral converges,

27 $$\begin{aligned} \frac{\ln \tau ^{(N)}}{N}\rightarrow &  \int _{\Gamma _0} \varvec{\theta }( {\varvec{y}}^\prime ) \cdot d {\varvec{y}}^\prime \text{ as } N \rightarrow \infty , \end{aligned}$$

where $\Gamma _0$ is any path from ${\varvec{y}}^*$ to $\textbf{0}$ that lies entirely within ${\tilde{S}}$, and the integral is independent of the particular path $\Gamma _0$.

#### Result 5

Suppose that conditions (24) and (25) hold. With $\varvec{\theta }( {\varvec{y}})$ denoting the solution of equations (26), define the matrix $\Sigma$ to be

28 $$\begin{aligned} \Sigma= &  \left. \frac{\partial \varvec{\theta }}{\partial {\varvec{y}}} \right| _{{\varvec{y}}= {\varvec{y}}^*}. \end{aligned}$$

Suppose that for $a_1, a_2, \ldots , a_n \in {{\mathbb {Z}}}$ and ${\varvec{l}}_1, {\varvec{l}}_2, \ldots , {\varvec{l}}_n \in {{\mathcal {L}}}$ we have

29 $$\begin{aligned} \sum _{i=1}^n a_i {\varvec{l}}_i = \textbf{0}\Rightarrow &  \sum _{i=1}^n a_i {\varvec{l}}_i^T \frac{\partial }{\partial {\varvec{y}}} \ln \left( \beta _{-\varvec{l}_i}( \varvec{y} ) \beta _{\varvec{l}_i}( \varvec{y} ) \right) = 0 \text{ for } \text{ all } {\varvec{y}}\in \tilde{S}^\circ , \end{aligned}$$

and that for every ${\varvec{l}}_1, {\varvec{l}}_2 \in {{\mathcal {L}}}$,

30 $$\begin{aligned} {\varvec{l}}_1^T \left( \frac{\partial ^2}{\partial {\varvec{y}}^2} \ln \left( \beta _{-{\varvec{l}}_1} ( {\varvec{y}}) \beta _{{\varvec{l}}_1} ( {\varvec{y}}) \right) \right) {\varvec{l}}_2= &  {\varvec{l}}_2^T \left( \frac{\partial ^2}{\partial {\varvec{y}}^2} \ln \left( \beta _{-{\varvec{l}}_2} ( {\varvec{y}}) \beta _{{\varvec{l}}_2} ( {\varvec{y}}) \right) \right) {\varvec{l}}_1. \end{aligned}$$

Then the system of equations

31 $$\begin{aligned} {\varvec{l}}^T \varvec{\theta }^{0} ( {\varvec{y}})= &  \frac{1}{2} {\varvec{l}}^T \frac{\partial }{\partial {\varvec{y}}} \ln \left( \beta _{-{\varvec{l}}} ( {\varvec{y}}) \beta _{{\varvec{l}}} ( {\varvec{y}}) \right) \text{ for } \text{ all } {\varvec{l}}\in {{\mathcal {L}}} \end{aligned}$$

has a unique solution $\varvec{\theta }^{0} ( {\varvec{y}})$ for each ${\varvec{y}}\in \tilde{S}^\circ$, and the body of the quasistationary distribution may be approximated as follows. Writing ${\varvec{y}}= {\varvec{x}}/ N$, for ${\varvec{y}}\in {\tilde{S}}^\circ$ with $d ( {\varvec{y}}, \partial {\tilde{S}} ) \ne o(1)$, then ${\varvec{y}}$ may be expressed (non-uniquely) as ${\varvec{y}}= {\varvec{y}}^* + \sum _{i=1}^n a_i {\varvec{l}}_i$ for some $a_1, a_2, \ldots , a_n \in {{\mathbb {R}}}$ and ${\varvec{l}}_1, {\varvec{l}}_2, \ldots , {\varvec{l}}_n \in {{\mathcal {L}}}$ such that ${\varvec{y}}^* + \sum _{i=1}^j a_i {\varvec{l}}_i \in {\tilde{S}}$ for $j=1,2,\ldots ,n$, and we then have

32 $$\begin{aligned} u_{{\varvec{x}}}^{(N)}= &  \sqrt{\frac{\text{ det } ( \Sigma )}{(2 \pi N)^k} \prod _{j=1}^n \frac{\beta _{-{\varvec{l}}_j} \left( {\varvec{y}}^* + \sum _{i=1}^{j-1} a_i {\varvec{l}}_i \right) \beta _{{\varvec{l}}_j} \left( {\varvec{y}}^* + \sum _{i=1}^{j-1} a_i {\varvec{l}}_i \right) }{\beta _{-{\varvec{l}}_j} \left( {\varvec{y}}^* + \sum _{i=1}^{j} a_i {\varvec{l}}_i \right) \beta _{{\varvec{l}}_j} \left( {\varvec{y}}^* + \sum _{i=1}^{j} a_i {\varvec{l}}_i \right) }} \nonumber \\  &  {} \times \exp \left( - N \int _{\Gamma } \varvec{\theta }( {\varvec{y}}^\prime ) \cdot d {\varvec{y}}^\prime + O (1/N) \right) \end{aligned}$$

where $\Gamma$ is any path from ${\varvec{y}}^*$ to ${\varvec{y}}$ that lies entirely within ${\tilde{S}}$, the integral is independent of the particular path $\Gamma$, and the formula (32) is independent of the particular representation ${\varvec{y}}= {\varvec{y}}^* + \sum _{i=1}^n a_i {\varvec{l}}_i$ chosen.

#### Remark 5

If the process ${\varvec{X}}^{(N)} (t)$ satisfies the Kolmogorov criterion (9) for all *N*, then conditions (24), (25), (29), (30) are all satisfied. This may be seen by substituting the density dependent form of the transition rates from equation (1) into the relationship (9) and expanding in powers of *N*. We find that leading order terms correspond to condition (24). Second order terms around parallelograms (small loops of the form ${\varvec{x}}\rightarrow {\varvec{x}}+ {\varvec{l}}_1 \rightarrow {\varvec{x}}+ {\varvec{l}}_1 + {\varvec{l}}_2 \rightarrow {\varvec{x}}+ {\varvec{l}}_2 \rightarrow {\varvec{x}}$) correspond to condition (25), or condition (15) in the case of multitype birth and death processes. Second order terms around more general closed paths imply condition (29). Third order terms around parallelograms correspond to condition (30), or condition (19) for multitype birth and death processes.

On the other hand, if we substitute the density dependent form of the transition rates from equation (1), together with the WKB form (4) for the quasistationary distribution ${\varvec{u}}^{(N)}$, into the detailed balance equations (10) and expand in powers of *N*, we find that leading order terms correspond to equation (26) with $\varvec{\theta }= \frac{\partial V}{\partial {\varvec{y}}}$, and that second order terms then correspond to equation (31) with $\varvec{\theta }^0 = \frac{\partial V_0}{\partial {\varvec{y}}}$. The key to our derivations (section 6) is that asymptotic versions of the Kolmogorov criterion imply corresponding asymptotic versions of the detailed balance equations, which in turn allow explicit solution of the Hamilton-Jacobi equation (5) for $V ( {\varvec{y}})$ and of the transport equation (6) for $V_0 ( {\varvec{y}})$.

We discuss these issues further, and the relationship with concepts more familiar from vector calculus, in remark 6 at the end of section 6.

## Applications

We defer derivations to section 6, and first illustrate our results in some specific applications. Examples 1 and 2 below are specific models for which assumptions 1–8, as well as conditions (13), (15), (19) and (21), may be straightforwardly verified. Examples 3 and 4, on the other hand, consist of general classes of processes, and here we leave assumptions 7 and 8 to be verified in any specific case of interest.

### Example 1

*The susceptible-infectious-susceptible (SIS) infection model with heterogeneous susceptibilities and infectious periods of* Clancy (2018b). This process has finite state space $S^{(N)} = \left\{ {\varvec{x}}= \left( x_ 1, x_2, \ldots , x_k \right) : 0 \le x_i \le N_i \text{ for } i=1,2,\ldots ,k \right\}$, where $N_i$ represents the (constant) total number of type *i* individuals in the population, with $N_1 + N_2 + \cdots + N_k = N$, and $X_i^{(N)} (t)$ is the number of infectious type *i* individuals at time *t*. In order that assumption 1 is satisfied, we require that $f_i = N_i / N > 0$ for $i=1,2,\ldots ,k$, and that $f_1, f_2, \ldots , f_k$ do not vary with *N*. For this model, ${{\mathcal {L}}} = \{ {\varvec{e}}_i, - {\varvec{e}}_i: i = 1,2,\ldots ,k \}$, with transition rates given by equation (1) with $\beta _{{\varvec{e}}_i} ({\varvec{y}}) = \beta \mu _i \left( f_i - y_i \right) \sum _{j=1}^k y_j$ and $\beta _{-{\varvec{e}}_i} ({\varvec{y}}) = y_i / \alpha _i$. Here $\beta > 0$ is an overall infection rate parameter, while for $i=1,2,\ldots ,k$, $\mu _i > 0$ gives the level of susceptibility of uninfected type *i* individuals and $\alpha _i > 0$ is the mean infectious period of infected type *i* individuals. Without loss of generality the $\mu _i$ values are scaled so that $\sum _{i=1}^k \mu _i f_i = 1$. Assumptions 2–6 are straightforward to check. In order that assumption 7 is satisfied, we require $\beta \sum _{i=1}^k \alpha _i \mu _i f_i > 1$,  (Clancy 2018b; Lajmanovich and Yorke 1976). Since the state space is finite, assumption 8 is satisfied (Darroch and Seneta 1967).

From the definitions (12) we have $b_{ij} = \beta \mu _i f_i$ and $d_i = 1 / \alpha _i$ for $i,j=1,2,\ldots ,k$, so that conditions (13) and (21) are satisfied. Denoting by *E* the unique positive solution of

$$\begin{aligned} \beta \sum _{i=1}^k \frac{\alpha _i \mu _i f_i}{\alpha _i \mu _i E + 1}= &  1, \end{aligned}$$

then from equation (9) of Clancy (2018b), the stable equilibrium point ${\varvec{y}}^*$ has components

$$\begin{aligned} y_i^*= &  \frac{\alpha _i \mu _i f_i E}{1 + \alpha _i \mu _i E} \text{ for } i=1,2,\ldots ,k. \end{aligned}$$

It is straightforward to check that conditions (15) and (19) are satisfied. Following some algebraic manipulation, including applying the matrix determinant lemma to evaluate $\text{ det } ( \Sigma )$, the relationship (23) reduces to

$$\begin{aligned} \tau ^{(N)}\sim &  \frac{1}{DE} \sqrt{\frac{2\pi }{N} \left( \sum _{i=1}^k f_i \left( \frac{\alpha _i \mu _i}{1 + \alpha _i \mu _i E} \right) ^2 \right) ^{-1}} \; \exp \left( N \left( \sum _{i=1}^k f_i \ln \left( 1 + \alpha _i \mu _i E \right) - \frac{E}{\beta } \right) \right) , \end{aligned}$$

in agreement with formula (6) of Clancy (2018b) for the case of exponentially distributed infectious periods.

### Example 2

*The multitype birth and death process with linear birth rates and quadratic death rates of* Chazottes et al. (2019), *Section 2.2.* This process has state space $S^{(N)} = {{\mathbb {Z}}}_+^k$ for all *N*, and $\mathcal{L} =\, \{ {\varvec{e}}_i, - {\varvec{e}}_i: i = 1,2,\ldots ,k \}$, with transition rates given by equation (1) with $\beta _{{\varvec{e}}_i} ( {\varvec{y}}) = \lambda \sum _{j=1}^k y_j$ and $\beta _{-{\varvec{e}}_i} ( {\varvec{y}}) = y_i \left( \mu + \kappa \sum _{j=1}^k y_j \right)$ for parameters $\lambda , \mu , \kappa > 0$. Assumptions 1–6 are clearly satisfied. In order that assumption 7 is satisfied, we require $k \lambda > \mu$, see Ball and Clancy (2023); Chazottes et al. (2019). Assumption 8 then follows from Chazottes et al. (2019). It is straightforward to check that conditions (13), (15), (19) and (21) are satisfied, and that the relationship (23) reduces, after some algebraic manipulation, to

33 $$\begin{aligned} \tau ^{(N)}\sim &  \frac{1}{(k \lambda - \mu )^2} \sqrt{\frac{2 \pi \mu \kappa }{N}} \exp \left( N \left( \frac{k\lambda - \mu }{\kappa } + \frac{\mu }{\kappa }\ln \left( \frac{\mu }{k \lambda } \right) \right) \right) . \end{aligned}$$

This is in agreement with formula (49) and equations (51) and (52) of Ball and Clancy (2023), with the probability $\omega$ in equation (52) of Ball and Clancy (2023) being given by $\omega = \mu / k \lambda$, corresponding to the formula immediately before equation (55) of Ball and Clancy (2023).

### Example 3

*The multitype birth and death processes of* Ball and Clancy (2023). Here, we take the state space $S^{(N)}$ to be either the whole of ${{\mathbb {Z}}}_+^k$ for all *N*, or the finite set $S^{(N)} = \left\{ {\varvec{x}}= \left( x_ 1, x_2, \ldots , x_k \right) : 0 \le x_i \le N_i \text{ for } i=1,2,\ldots ,k \right\}$ for some ${\varvec{N}}= \left( N_1, N_2, \ldots , N_k \right)$ with $N_1 + N_2 + \cdots + N_k = N$, and in the case of finite state space, writing $f_i = N_i / N$ for $i = 1,2, \ldots ,k$, we require that $f_1, f_2, \ldots , f_k$ do not vary with *N*, and that $f_1, f_2, \ldots , f_k > 0$. Thus assumption 1 is satisfied. Taking ${{\mathcal {L}}} = \{ {\varvec{e}}_i, - {\varvec{e}}_i: i=1,2,\ldots ,k \}$, and with transition rates given by equation (1) with $\beta _{{\varvec{e}}_i} ( {\varvec{y}}) = b_0 \left( \sum _{j=1}^k y_j \right) b_i ( y_i )$ and $\beta _{-{\varvec{e}}_i} ( {\varvec{y}}) = d_0 \left( \sum _{j=1}^k y_j \right) d_i ( y_i )$ for appropriate functions $b_0, b_1, \ldots , b_k, d_0, d_1, \ldots , d_k$ from ${{\mathbb {R}}}_+$ to ${{\mathbb {R}}}_+$, we obtain the form of multitype birth and death process considered by Ball and Clancy (2023). Note that assumptions 2 and 3 are satisfied.

In the case that $S^{(N)} = {{\mathbb {Z}}}_+^k$ for all *N*, denote ${\tilde{C}}_0 = {\tilde{C}}_1 = \ldots = {\tilde{C}}_k = ( 0, \infty )$, while in the case that $S^{(N)}$ is finite, denote ${\tilde{C}}_0 = (0,1)$ and ${\tilde{C}}_i = ( 0, f_i )$ for $i=1,2,\ldots ,k$. We require that $b_0 (0) = 0$ and that for $i=1,2,\ldots ,k$, $b_i (0) > 0$ and $d_i (0) = 0$. For $i=0,1,\ldots ,k$ and $y \in {\tilde{C}}_i$ we require that $b_i ( y ) > 0$ and $d_i (y) > 0$. In the case of finite state space, we additionally require that $d_0 (1) > 0$ and that for $i=1,2,\ldots ,k$, $b_i ( f_i ) = 0$ and $d_i ( f_i ) > 0$. These requirements, corresponding to assumptions (4)–(6) of Ball and Clancy (2023), ensure that our assumptions 4 and 5 are satisfied. In order that assumption 6 is satisfied, we require that for $i=0,1,\ldots ,k$, the functions $b_i$ and $d_i$ are twice differentiable throughout their domains, corresponding roughly to assumptions (7) and (37)–(39) of Ball and Clancy (2023). From our requirements (above) that $b_0 (0) = 0$ and $d_i (0) = 0$ for $i=1,2,\ldots ,k$, it follows that the system (11) has an equilibrium point at ${\varvec{y}}= \textbf{0}$. To ensure that the equilibrium point at ${\varvec{y}}= \textbf{0}$ is locally unstable, we require (see Ball and Clancy 2023) that

$$\begin{aligned} \frac{b_0^\prime (0)}{d_0(0)} \sum _{i=1}^k \frac{b_i(0)}{d_j^\prime (0)} > 1. \end{aligned}$$

In order that assumption 7 is satisfied, we further require the functions $b_0, b_1, \ldots , b_k, d_0, d_1, \ldots , d_k$ to be such that there exists precisely one other equilibrium point ${\varvec{y}}^* \in {\tilde{S}}$ of the system (11), that ${\varvec{y}}^* \in {\tilde{S}}^\circ$, and that all eigenvalues of the Jacobian of (11) at ${\varvec{y}}^*$ have strictly negative real part. These requirements are generally straightforward to check in specific applications, and correspond to assumptions (35) and (36) of Ball and Clancy (2023). In the case of finite state space, assumption 8 is automatically satisfied (Darroch and Seneta 1967). In the case of infinite state space, as previously remarked, checking assumption 8 can be far from trivial; we simply assume here that the functions $b_0, b_1, \ldots , b_k, d_0, d_1, \ldots , d_k$ are such that assumption 8 is indeed satisfied.

In order that condition (13) is satisfied, we require that $d_0 (0) > 0$, $b_0^\prime (0) > 0$, and for $i=1,2,\ldots ,k$, $d_i^\prime (0) > 0$, corresponding to assumption (8) of Ball and Clancy (2023). It is then straightforward to check that conditions (15), (19) and (21) are satisfied, and that the relationship (23) reduces, after some algebraic manipulation, including use of the matrix determinant lemma to evaluate $\text{ det } ( \Sigma )$, to the result given by formula (49) together with equations (41), (42) and (45) of Ball and Clancy (2023).

### Example 4

*Competition processes.* Taking $k=2$ and ${{\mathcal {L}}} = \{ {\varvec{e}}_1, - {\varvec{e}}_1, {\varvec{e}}_2, - {\varvec{e}}_2, {\varvec{e}}_2 - {\varvec{e}}_1, {\varvec{e}}_1 - {\varvec{e}}_2 \}$ corresponds to the ‘competition processes’ of Reuter (1961). We consider here a particular class of competition processes. We take the state space $S^{(N)}$ to be either the whole of ${{\mathbb {Z}}}_+^k$ for all *N*, or the finite set $S^{(N)} = \left\{ {\varvec{x}}= \left( x_ 1, x_2, \ldots , x_k \right) : 0 \le x_i \le N_i \text{ for } i=1,2,\ldots ,k \right\}$ for some ${\varvec{N}}= \left( N_1, N_2, \ldots ,\right. \left. N_k \right)$ with $N_1 + N_2 + \cdots + N_k = N$, and in the case of finite state space, writing $f_i = N_i / N$ for $i = 1,2, \ldots ,k$, we require that $f_1, f_2, \ldots , f_k$ do not vary with *N*, and that $f_1, f_2, \ldots , f_k > 0$. Thus assumption 1 is satisfied. We take transition rates to be of the form given by equation (1), with

34 $$\begin{aligned} \left. \begin{array}{rcl} \beta _{-{\varvec{e}}_1} ( {\varvec{y}}) & =& a_1 d_0 ( y_1 + y_2 ) d_1 ( y_1 ) c_2 ( y_2 ) d_3 ( y_1 - y_2 ), \\ \beta _{{\varvec{e}}_1} ( {\varvec{y}}) & =& a_2 b_0 ( y_1 + y_2 ) b_1 ( y_1 ) c_2 ( y_2 ) b_3 ( y_1 - y_2 ), \\ \beta _{-{\varvec{e}}_2} ( {\varvec{y}}) & =& a_3 d_0 ( y_1 + y_2 ) c_1 ( y_1 ) d_2 ( y_2 ) b_3 ( y_1 - y_2 ), \\ \beta _{{\varvec{e}}_2} ( {\varvec{y}}) & =& a_4 b_0 ( y_1 + y_2 ) c_1 ( y_1 ) b_2 ( y_2 ) d_3 ( y_1 - y_2 ), \\ \beta _{{\varvec{e}}_1 - {\varvec{e}}_2} ( {\varvec{y}}) & =& a_5 c_0 ( y_1 + y_2 ) b_1 ( y_1 ) d_2 ( y_2 ) b_3^2 ( y_1 - y_2 ), \\ \beta _{{\varvec{e}}_2 - {\varvec{e}}_1} ( {\varvec{y}}) & =& a_6 c_0 ( y_1 + y_2 ) d_1 ( y_1 ) b_2 ( y_2 ) d_3^2 ( y_1 - y_2 ), \end{array} \right\} \end{aligned}$$

for some positive constants $a_1, a_2, a_3, a_4, a_5, a_6$, functions $b_0, b_1, b_2, c_0, c_1, c_2, d_0, d_1, d_2$ from ${{\mathbb {R}}}_+$ to ${{\mathbb {R}}}_+$, and functions $b_3, d_3$ from ${{\mathbb {R}}}$ to ${{\mathbb {R}}}_+$. Thus assumptions 2 and 3 are satisfied.

In the case that $S^{(N)} = {{\mathbb {Z}}}_+^k$ for all *N*, denote ${\tilde{C}}_0 = {\tilde{C}}_1 = {\tilde{C}}_2 = ( 0, \infty )$ and ${\tilde{C}}_3 = ( - \infty , \infty )$. In the case that $S^{(N)}$ is finite, denote ${\tilde{C}}_0 = (0,1)$, ${\tilde{C}}_1 = ( 0, f_1 )$, ${\tilde{C}}_2 = ( 0, f_2 )$ and ${\tilde{C}}_3 = ( -f_2, f_1 )$. In order that assumptions 4 and 5 are satisfied, we require that $b_0(0) = d_1 (0) = d_2 (0) = 0$, that $b_1 (0), b_2 (0), c_1 (0), c_2 (0) > 0$, that for $i=0,1,2,3$, we have $b_i(y) , d_i(y) > 0$ for $y \in {\tilde{C}}_i$, and that for $i=0,1,2$, we have $c_i (y) > 0$ for $y \in {\tilde{C}}_i$. In the case of finite state space, we additionally require that $b_1 ( f_1 ) = b_2 ( f_2 ) = 0$, and that $b_3 ( - f_2 ) , c_1 ( f_1 ) , c_2 ( f_2 ) , d_0 ( 1 ) , d_1 ( f_1 ) , d_2 ( f_2 ) , d_3 ( f_1 ) > 0$. In order that assumption 6 is satisfied, we require that the functions $b_i$ and $d_i$ for $i=0,1,2,3$, and $c_i$ for $i=0,1,2$, are all twice differentiable throughout their domains.

From our requirements (above) that $b_0(0) = d_1(0) = d_2 (0) = 0$ it follows that the system (11) has an equilibrium point at ${\varvec{y}}= \textbf{0}$. In order that assumption 7 is satisfied, we require the constants $a_1, a_2, a_3, a_4, a_5, a_6$ and functions $b_0, b_1, b_2, b_3, c_0, c_1, c_2, d_0, d_1, d_2, d_3$ to be such that the equilibrium point of the system (11) at ${\varvec{y}}= \textbf{0}$ is locally unstable; that there exists precisely one other equilibrium point ${\varvec{y}}^* \in {\tilde{S}}$; that ${\varvec{y}}^* \in \tilde{S}^\circ$; and that all eigenvalues of the Jacobian of (11) at ${\varvec{y}}^*$ have strictly negative real part. These requirements are generally straightforward to check in specific applications. In the case of finite state space, assumption 8 is automatically satisfied (Darroch and Seneta 1967); in the case of infinite state space, as in example 3, we simply assume that the constants $a_1, a_2, a_3, a_4, a_5, a_6$ and functions $b_0, b_1, b_2, b_3, c_0, c_1, c_2, d_0, d_1, d_2, d_3$ are such that assumption 8 is satisfied.

The role of condition (24) is to ensure that there exists a (unique) solution to equations (26) (see derivation of result 4 in section 6.1). It is more straightforward here to check directly that a unique solution to equations (26) exists, as follows. Equations (26) in this case are

35 $$\begin{aligned} \theta _1 ( {\varvec{y}})= &  \ln \left( \frac{a_1 d_0 ( y_1 + y_2 ) d_1 ( y_1 ) d_3 ( y_1 - y_2 )}{a_2 b_0 ( y_1 + y_2 ) b_1 ( y_1 ) b_3 ( y_1 - y_2 )} \right) , \end{aligned}$$

36 $$\begin{aligned} \theta _2 ( {\varvec{y}})= &  \ln \left( \frac{a_3 d_0 ( y_1 + y_2 ) d_2 ( y_1 ) b_3 ( y_1 - y_2 )}{a_4 b_0 ( y_1 + y_2 ) b_2 ( y_2 ) d_3 ( y_1 - y_2 )} \right) , \end{aligned}$$

37 $$\begin{aligned} \theta _1 ( {\varvec{y}}) - \theta _2 ( {\varvec{y}})= &  \ln \left( \frac{a_6 d_1 ( y_1 ) b_2 ( y_2 ) d_3^2 ( y_1 - y_2 )}{a_5 b_1 ( y_1 ) d_2 ( y_2 ) b_3^2 ( y_1 - y_2 )} \right) . \end{aligned}$$

Equations (35) and (36) give $\theta _1 ( {\varvec{y}})$, $\theta _2 ( {\varvec{y}})$ explicitly, and subtracting equation (36) from equation (35), we see that equations (35)–(37) can be simultaneously satisfied precisely when $\ln ( a_1 a_4 / a_2 a_3 ) = \ln ( a_6 / a_5 )$. That is, we require $a_1 a_4 a_5 = a_2 a_3 a_6$. It is then straightforward to check that condition (25) is satisfied.

With $\varvec{\theta }_1 ( {\varvec{y}}), \varvec{\theta }_2 ( {\varvec{y}})$ given by equations (35) and (36), respectively, the function $V ( {\varvec{y}})$ given by equation (52) may be written as

38 $$\begin{aligned} V ( y_1, y_2 )= &  ( y_1^* - y_1 ) \ln \left( \frac{a_2}{a_1} \right) + ( y_2^* - y_2 ) \ln \left( \frac{a_4}{a_3} \right) + \int _{y_1 + y_2}^{y_1^* + y_2^*} \ln \left( \frac{b_0(u)}{d_0(u)} \right) du \nonumber \\  &  {} \hspace{-20mm} + \int _{y_1}^{y_1^*} \ln \left( \frac{b_1(u)}{d_1(u)} \right) du + \int _{y_2}^{y_2^*} \ln \left( \frac{b_2(u)}{d_2(u)} \right) du + \int _{y_1 - y_2}^{y_1^* - y_2^*} \ln \left( \frac{b_3(u)}{d_3(u)} \right) du. \end{aligned}$$

In light of equation (52), the relationship (27) reduces to $\left( \ln \tau ^{(N)} \right) / N \rightarrow V ( 0,0 )$ as $N \rightarrow \infty$ with $V ( y_1, y_2 )$ given by equation (38), provided the integrals in equation (38) with $y_1 = y_2 = 0$ all converge. A sufficient condition for these integrals to converge is that the limits $\lim _{u \downarrow 0} b_0 (u) / u d_0 (u)$, $\lim _{u \downarrow 0} u b_1 (u) / d_1 (u)$ and $\lim _{u \downarrow 0} u b_2 (u) / d_2 (u)$ all exist and are finite. To see this, first observe that our requirements that $b_3 (u) , d_3 (u) > 0$ for $u \in {\tilde{C}}_3$ and that ${\varvec{y}}^* \in {\tilde{S}}^\circ$ together imply that the integrand $\ln \left( b_3 (u) / d_3 (u) \right)$ is bounded on the finite interval $\left[ 0, y_1^* - y_2^* \right]$, so that the corresponding integral in equation (38) is finite. For the other three integrals in equation (38), we argue as follows. Consider $\int _0^{y_2^*} \ln \left( b_2 (u) / d_2 (u) \right) du$. Since $b_2(0) > 0$ and $d_2 (0) = 0$, the integrand diverges at the origin. However, we have

39 $$\begin{aligned} \int _0^{y_2^*} \ln \left( \frac{b_2(u)}{d_2(u)} \right) du= &  \int _0^{y_2^*} \ln \left( \frac{b_2(u)}{u d_2(u)} \right) du + \int _0^{y_2^*} \ln u \, du \nonumber \\= &  \int _0^{y_2^*} \ln \left( \frac{b_2(u)}{u d_2(u)} \right) du + y_2^* \ln y_2^* - y_2^*. \end{aligned}$$

Now since $b_2 (u) , d_2 (u) > 0$ for $u \in {\tilde{C}}_2$, and ${\varvec{y}}^* \in {\tilde{S}}^{\circ }$, then provided $\lim _{u \downarrow 0} b_2 (u) / u d_2 (u)$ is finite, the integral on the right hand side of equation (39) converges, as required. We argue similarly for the integrals involving $b_1, d_1$ and $b_0, d_0$ in equation (38).

To check condition (29), as in the case of condition (24) above, it is more straightforward to check directly that a unique solution to equations (31) exists. We find that this is the case provided that $b_3 (u) d_3 (u) = a_0$ for all $u \in {{\mathbb {R}}}$ for some constant $a_0 > 0$. It is then straightforward to check that condition (30) is satisfied, and with ${\varvec{y}}= {\varvec{x}}/N$, equation (32) may be written as

40 $$\begin{aligned} u_{{\varvec{x}}}^{(N)}= &  \sqrt{\frac{\text{ det }(\Sigma )}{(2 \pi N)^2} \left( \frac{b_0 ( y_1^* + y_2^* ) d_0 ( y_1^* + y_2^* ) b_1 ( y_1^* ) d_1 ( y_1^* ) b_2 ( y_2^* ) d_2 ( y_2^* )}{b_0 ( y_1 + y_2 ) d_0 ( y_1 + y_2 ) b_1 ( y_1 ) d_1 ( y_1 ) b_2 ( y_2 ) d_2 ( y_2 )} \right) } \nonumber \\  &  {} \times \exp \left( - N V ( y_1, y_2 ) + O (1/N)\right) \end{aligned}$$

for $d ( {\varvec{y}}, \partial {\tilde{S}} ) \ne o(1)$, where $V(y_1, y_2)$ is given by equation (38), and the determinant is given by

41 $$\begin{aligned} \text{ det } ( \Sigma )= &  4 \left( \frac{d_0^\prime (y_1^* + y_2^*)}{d_0 ( y_1^* + y_2^* )} - \frac{b_0^\prime ( y_1^* + y_2^*)}{b_0 ( y_1^* + y_2^* )} \right) \left( \frac{d_3^\prime ( y_1^* - y_2^* )}{d_3 ( y_1^* - y_2^* )} - \frac{b_3^\prime ( y_1^* - y_2^* )}{b_3 ( y_1^* - y_2^* )} \right) \nonumber \\  &  {} + \left( \frac{d_1^\prime ( y_1^* )}{d_1 ( y_1^* )} - \frac{b_1^\prime ( y_1^* )}{b_1 ( y_1^* )} \right) \left( \frac{d_2^\prime ( y_2^* )}{d_2 ( y_2^* )} - \frac{b_2^\prime ( y_2^* )}{b_2 ( y_2^* )} \right) \nonumber \\  &  {} + \left( \frac{d_0^\prime (y_1^* + y_2^*)}{d_0 ( y_1^* + y_2^* )} - \frac{b_0^\prime ( y_1^* + y_2^*)}{b_0 ( y_1^* + y_2^* )} + \frac{d_3^\prime ( y_1^* - y_2^* )}{d_3 ( y_1^* - y_2^* )} - \frac{b_3^\prime ( y_1^* - y_2^* )}{b_3 ( y_1^* - y_2^* )} \right) \nonumber \\  &  {} \times \left( \frac{d_1^\prime ( y_1^* )}{d_1 ( y_1^* )} - \frac{b_1^\prime ( y_1^* )}{b_1 ( y_1^* )} + \frac{d_2^\prime ( y_2^* )}{d_2 ( y_2^* )} - \frac{b_2^\prime ( y_2^* )}{b_2 ( y_2^* )} \right) . \end{aligned}$$

The expression on the right hand side of equation (41) may be slightly simplified by making use of the relationship $b_3 (u) d_3 (u) = a_0$, but we will require the more general form given in equation (41) in what follows.

Taking $a_5 = a_6 = 0$, and correspondingly redefining ${{\mathcal {L}}} = \{ {\varvec{e}}_1, - {\varvec{e}}_1, {\varvec{e}}_2, - {\varvec{e}}_2 \}$, the transition rate functions (34) are now of the form of the ‘reversible competition processes’ of the corollary to theorem 5 of Iglehart (1964). From definitions (12) we find

42 $$\begin{aligned} \left. \begin{array}{rcl} b_{11} & =& a_2 b_0^\prime (0) b_1(0) c_2(0) b_3(0), \\ b_{22} & =& a_4 b_0^\prime (0) c_1(0) b_2(0) d_3(0), \\ d_1 & =& a_1 d_0(0) d_1^\prime (0) c_2(0) d_3(0), \\ d_2 & =& a_3 d_0(0) c_1(0) d_2^\prime (0) b_3(0). \end{array} \right\} \end{aligned}$$

In order that condition (13) is satisfied, we require $d_0(0), b_0^\prime (0), d_1^\prime (0), d_2^\prime (0) > 0$. It is straightforward to check that conditions (15), (19) and (21) are satisfied. Note that the constraint $b_3(u) d_3(u) = a_0$ is no longer required. The relationship (23) now reduces to

43 $$\begin{aligned} \tau ^{(N)}\sim &  \frac{1}{D} \sqrt{ \frac{2 \pi }{N \text{ det } ( \Sigma )} \frac{b_0^\prime (0) d_0(0) b_1(0) d_1^\prime (0) b_2(0) d_2^\prime (0) b_3(0) d_3(0)}{b_0 ( y_1^* + y_2^* ) d_0 ( y_1^* + y_2^* ) b_1 ( y_1^* ) d_1 ( y_1^* ) b_2 ( y_2^* ) d_2 ( y_2^* ) b_3 ( y_1^* - y_2^* ) d_3 ( y_1^* - y_2^* )} } \nonumber \\  &  {} \times \exp ( N V (0,0) ), \end{aligned}$$

where $\text{ det } ( \Sigma )$ is given by equation (41), *D* is the unique positive solution of equation (22) with $b_{11}, b_{22}, d_1, d_2$ given by equations (42), and the function $V ( y_1, y_2 )$ is given by equation (38). Condition (13) ensures that the integrals in equation (38) with $y_1 = y_2 = 0$ all converge.

Note that it is straightforward in principle to generalise example 4 to $k>2$ dimensions, but the notation becomes very cumbersome.

## Numerical illustration

For numerical illustration, we consider a specific model within the framework of example 4. Thus we have $k=2$ and ${{\mathcal {L}}} = \{ {\varvec{e}}_1, - {\varvec{e}}_1, {\varvec{e}}_2, - {\varvec{e}}_2, {\varvec{e}}_2 - {\varvec{e}}_1, {\varvec{e}}_1 - {\varvec{e}}_2 \}$, and we take transition rates to be of the form given by equation (1) with, for $i, j \in \{ 1,2 \}$ and $j \ne i$,

44 $$\begin{aligned} \left. \begin{array}{rcl} \beta _{-{\varvec{e}}_i} ( y_1, y_2 ) & =& y_i \left( \mu + \kappa ( y_1 + y_2 ) \right) , \\ \beta _{{\varvec{e}}_i} ( y_1, y_2 ) & =& \lambda _i ( y_1 + y_2 ), \\ \beta _{{\varvec{e}}_i - {\varvec{e}}_j} ( y_1, y_2 ) & =& \nu _j y_j, \end{array} \right\} \end{aligned}$$

for parameters $\mu , \kappa , \lambda _1, \lambda _2, \nu _1, \nu _2 > 0$. This represents a particular two group birth and death process incorporating movement of individuals between groups. In the case $\lambda _1 = \lambda _2$ and $\nu _1 = \nu _2 = 0$, this model reduces to the multitype birth and death process of example 2 in the case $k=2$.

Within the framework of example 4, the above model may be obtained by setting $a_1 = 1$, $a_2 = \lambda _1$, $a_3 = 1$, $a_4 = \lambda _2$, $a_5 = \nu _2$, $a_6 = \nu _1$, $b_0 (u) = u$, $b_1 (u) = 1$, $b_2 (u) = 1$, $b_3 (u) = 1$, $c_0 (u) = 1$, $c_1 (u) = 1$, $c_2 (u) = 1$, $d_0 (u) = \mu + \kappa u$, $d_1 (u) = u$, $d_2 (u) = u$, $d_3 (u) = 1$.

It is clear that assumptions 1–6 hold here. It is straightforward to show that assumption 7 holds provided $\lambda _1 + \lambda _2 > \mu$, and that the equilibrium point ${\varvec{y}}^* = \left( y_1^*, y_2^* \right)$ is then given by

45 $$\begin{aligned} \left( y_1^*, y_2^* \right)= &  \frac{\lambda _1 + \lambda _2 - \mu }{\kappa ( \lambda _1 + \lambda _2 + \nu _1 + \nu _2 )} \left( \lambda _1 + \nu _2, \lambda _2 + \nu _1 \right) . \end{aligned}$$

Since the state space is infinite, and the process does not fall within the framework of Chazottes et al. (2019), establishing the validity of assumption 8 is beyond the scope of this paper, and we shall simply proceed with numerical work.

We saw in example 4 that condition (24) is satisfied provided $a_1 a_4 a_5 = a_2 a_3 a_6$. For the current model, this condition becomes $\lambda _1 \nu _1 = \lambda _2 \nu _2$, and we will require from now on that this condition holds. Similarly, we saw in example 4 that condition (29) is satisfied provided $b_3 (u) d_3 (u) = a_0$ for all $u \in {{\mathbb {R}}}$ for some constant $a_0 > 0$; for the current model, since $b_3 (u) d_3 (u) = 1$, this condition is satisfied.

Under the condition $\lambda _1 \nu _1 = \lambda _2 \nu _2$, the equilibrium point (45) may be expressed more simply as

46 $$\begin{aligned} \left( y_1^*, y_2^* \right)= &  \frac{\lambda _1 + \lambda _2 - \mu }{\kappa ( \lambda _1 + \lambda _2 )} \left( \lambda _1, \lambda _2 \right) . \end{aligned}$$

Equation (38) now simplifies to

47 $$\begin{aligned} V ( y_1, y_2 )= &  y_1 \ln \left( \frac{y_1}{\lambda _1} \right) + y_2 \ln \left( \frac{y_2}{\lambda _2} \right) + ( y_1 + y_2 ) \left( \ln \left( \frac{\mu + \kappa ( y_1 + y_2 )}{y_1 + y_2} \right) - 1 \right) \nonumber \\  &  {} + \frac{\mu }{\kappa } \ln \left( 1 + \frac{\kappa }{\mu } ( y_1 + y_2 ) \right) + \frac{\lambda _1 + \lambda _2 - \mu }{\kappa } + \frac{\mu }{\kappa } \ln \left( \frac{\mu }{\lambda _1 + \lambda _2} \right) , \end{aligned}$$

so that the relationship (27) becomes

48 $$\begin{aligned} \frac{\ln \tau ^{(N)}}{N}\rightarrow &  \frac{\lambda _1 + \lambda _2 - \mu }{\kappa } + \frac{\mu }{\kappa } \ln \left( \frac{\mu }{\lambda _1 + \lambda _2} \right) \text{ as } N \rightarrow \infty . \end{aligned}$$

The matrix $\Sigma$ defined by equation (28) is here given by

49 $$\begin{aligned} \Sigma= &  \frac{\kappa }{\lambda _1 + \lambda _2 - \mu } \left( \begin{array}{cc} \frac{\lambda _1 + \lambda _2}{\lambda _1} - \frac{\mu }{\lambda _1 + \lambda _1} & - \frac{\mu }{\lambda _1 + \lambda _1}\\ - \frac{\mu }{\lambda _1 + \lambda _1} & \frac{\lambda _1 + \lambda _2}{\lambda _2} - \frac{\mu }{\lambda _1 + \lambda _1} \end{array} \right) , \end{aligned}$$

and equation (41) simplifies to

$$\begin{aligned} \text{ det } ( \Sigma )= &  \frac{\kappa ^2 ( \lambda _1 + \lambda _2 )}{\lambda _1 \lambda _2 ( \lambda _1 + \lambda _2 - \mu )}. \end{aligned}$$

With ${\varvec{y}}= {\varvec{x}}/N$, equation (40) thus becomes

50 $$\begin{aligned} u_{{\varvec{x}}}^{(N)}= &  \frac{\lambda _1 + \lambda _2 - \mu }{2 \pi N \sqrt{\kappa y_1 y_2 ( y_1 + y_2 ) ( \mu + \kappa ( y_1 + y_2 ) )}} \exp \left( - N V ( y_1, y_2 ) + O (1/N)\right) \nonumber \\ \end{aligned}$$

for $d ( {\varvec{y}}, \partial {\tilde{S}} ) \ne o(1)$, where $V ( y_1, y_2 )$ is given by equation (47).

**Fig. 1:**
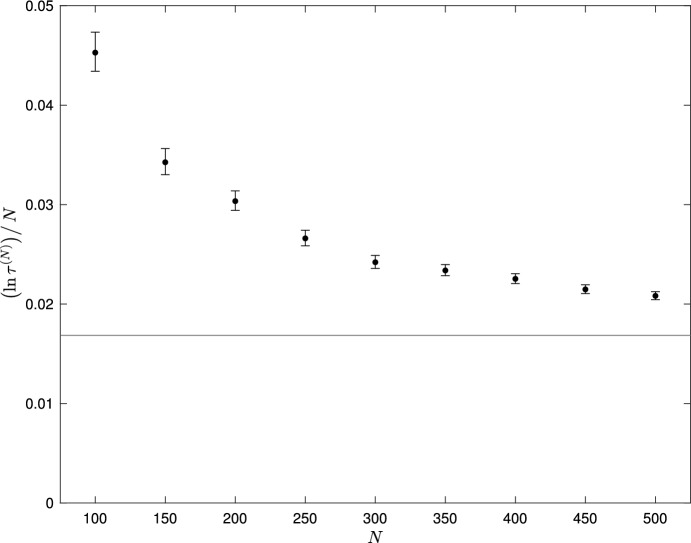
Plot of $\left( \ln \tau ^{(N)} \right) / N$ against *N* for the model with transition rates given by equations (44). Fixed parameter values $\mu = 2.9$, $\kappa = 0.1$, $\lambda _1 = 1$, $\lambda _2 = 2$, $\nu _1 = 2$, $\nu _2 = 1$. Simulation results based on 100 simulations for each value of *N*. with maximum likelihood estimates (dots) and 95% confidence intervals shown; horizontal line shows the value of *V*(0, 0) given by the right hand side of the relationship (48)

Figure 1 compares mean extinction times $\tau ^{(N)}$ estimated from Monte Carlo simulation with the asymptotic relationship (48), with parameter values $\mu = 2.9$, $\kappa = 0.1$, $\lambda _1 = 1$, $\lambda _2 = 2$, $\nu _1 = 2$, $\nu _2 = 1$, for a range of *N* values. Each simulation run was initiated at time $t=0$ at the point $\lceil N y_1^*, N y_2^* \rceil$ where $\left( y_1^*, y_2^* \right)$ is given by equation (46), and $\lceil x \rceil$ denotes the least integer greater than or equal to *x*. The simulation was first run for a burn-in period $t_0$, to allow the process to (approximately) attain quasistationarity, and the time from the end of the burn-in period until population extinction recorded. For each value of *N*, we ran 100 simulations; any runs that went extinct before the end of the burn-in period were re-started from $\lceil N y_1^*, N y_2^* \rceil$ at time $t=0$, to ensure a sample of 100 extinction times for each *N*. Simulations were run up to at most time $t_{max}$, so that recorded extinction times were right-censored at $t_{max} - t_0$. Since the time from quasistationarity to extinction is known to be exponentially distributed, the maximum likelihood estimate of $\tau ^{(N)}$ was then computed as described in section 6 of Clancy (2018a), and confidence intervals computed using formula (C6) of Sundberg (2001). Values of $t_0 = 10$ and $t_{max} = 50000$ were found to be sufficient to ensure that, for the parameter values of Figure 1, for each value of *N*, the sample of simulated extinction times was approximately exponentially distributed and a high proportion of simulation runs went extinct before time $t_{max}$. The horizontal line in Figure 1 indicates the value of *V*(0, 0) computed from the right hand side of the relationship (48). We see that as *N* increases, convergence of the empirically computed value of $\left( \ln \tau ^{(N)} \right) / N$ towards the theoretical value *V*(0, 0) is rather slow, similar to what was observed in Figure 3 of Clancy and Stewart (2024).

**Fig. 2:**
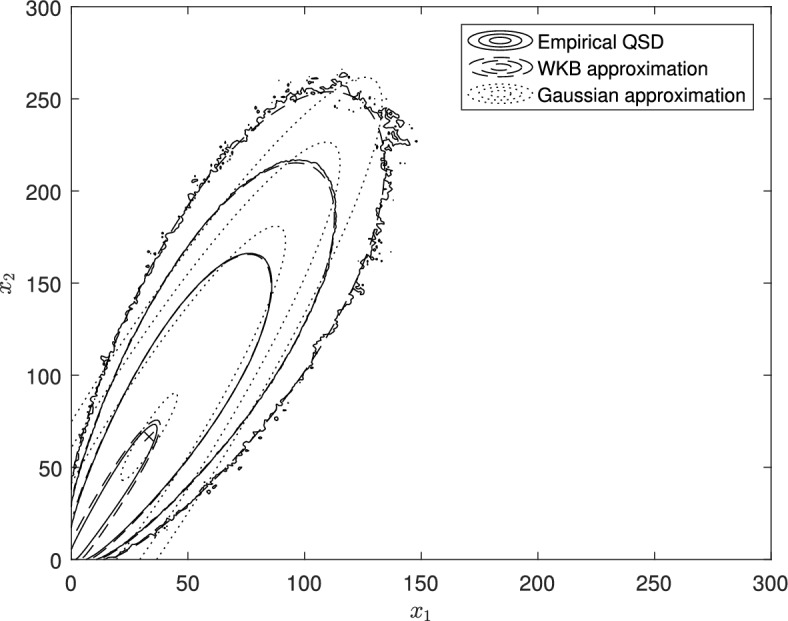
Contour plots of the empirical quasistationary distribution and approximations, for the model with transition rates given by equations (44). Parameter values $N=100$, $\mu = 2.9$, $\kappa = 0.1$, $\lambda _1 = 1$, $\lambda _2 = 2$, $\nu _1 = 2$, $\nu _2 = 1$. Contour levels correspond to probabilities $5 \times \left( 10^{-4},\, 10^{-6},\, 10^{-8},\, 10^{-10} \right)$. Solid contours represent the empirical quasistationary distribution computed from Monte Carlo simulation; dashed contours represent the WKB approximation (4); dotted contours represent the Gaussian approximation. Cross marks the deterministic equilibrium point $( N y_1^*, N y_2^* )$

Figure 2 shows contour plots of the empirically computed quasistationary distribution (solid lines), the WKB approximation (4) (dashed lines), and the bivariate normal approximation with mean $N {\varvec{y}}^*$ and variance matrix $N \Sigma ^{-1}$, where ${\varvec{y}}^* = \left( y_1^*, y_2^* \right)$ is given by equation (46) and $\Sigma$ is given by equation (49) (dotted lines). Parameter values are $N=100$, $\mu = 2.9$, $\kappa = 0.1$, $\lambda _1 = 1$, $\lambda _2 = 2$, $\nu _1 = 2$, $\nu _2 = 1$. The empirical quasistationary distribution was generated from 100000 simulation runs, each initiated at time $t=0$ at the point $\lceil N y_1^*, N y_2^* \rceil$, and run up to time $t_{max} = 50000$, or until extinction. Simulation runs that went extinct before the end of the burn-in period $t_0 = 10$ were re-started from $\lceil N y_1^*, N y_2^* \rceil$ at time $t=0$. The empirical quasistationary distribution $\widehat{\varvec{\pi }}$ was created by setting each component ${\hat{\pi }}_{{\varvec{x}}}$, for ${\varvec{x}}\ne \textbf{0}$, equal to the proportion of time between $t_0$ and $t_{max}$ spent in state ${\varvec{x}}$ by all non-extinct simulation runs. The WKB approximation is computed from formula (50), neglecting the *O*(1/*N*) term. We see from Figure 2 that the WKB approximation provides a very good approximation to the empirical quasistationary distribution, although the approximation becomes less accurate close to the origin, as expected. The bivariate Gaussian approximation is seen to be considerably less accurate than WKB approximation.

Taking $\nu _1 = \nu _2 = 0$, we can apply result 3 to this model. The relationship (43) reduces, after a little algebra, to

51 $$\begin{aligned} \hspace{-5mm} \tau ^{(N)}\sim &  \frac{1}{(\lambda _1 + \lambda _2 - \mu )^2} \sqrt{\frac{2 \pi \mu \kappa }{N}} \exp \left( N \left( \frac{\lambda _1 + \lambda _2 - \mu }{\kappa } + \frac{\mu }{\kappa }\ln \left( \frac{\mu }{\lambda _1 + \lambda _2} \right) \right) \right) .\nonumber \\ \end{aligned}$$

Note that in the case $\lambda _1 = \lambda _2$, the expression (51) agrees with expression (33) for the case $k=2$.

**Fig. 3:**
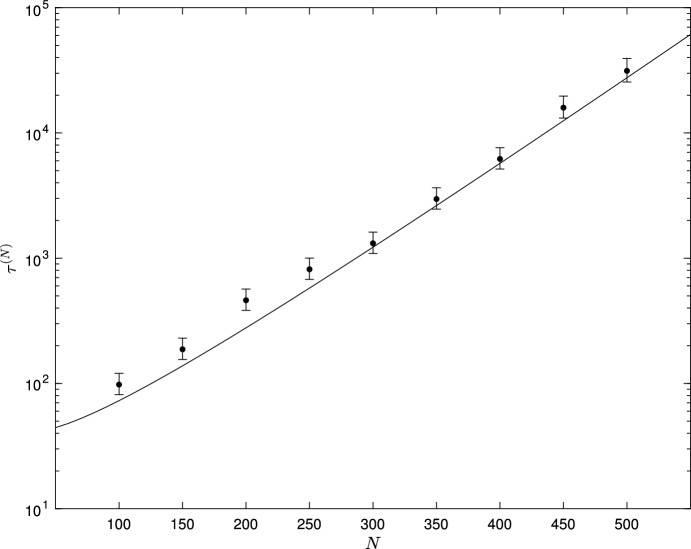
Plot of $\tau ^{(N)}$ (on a logarithmic scale) against *N* for the model with transition rates given by equations (44) in the case $\nu _1 = \nu _2 = 0$. Other fixed parameter values $\mu = 2.9$, $\kappa = 0.1$, $\lambda _1 = 1$, $\lambda _2 = 2$. Simulation results based on 100 simulations for each value of *N*. with maximum likelihood estimates (dots) and 95% confidence intervals shown; solid line shows the WKB asymptotic formula (51)

Figure 3 compares mean extinction times $\tau ^{(N)}$ estimated from Monte Carlo simulation for the case $\nu _1 = \nu _2 = 0$ with the asymptotic relationship (51), for a range of *N* values. Other parameter values are $\mu = 2.9$, $\kappa = 0.1$, $\lambda _1 = 1$, $\lambda _2 = 2$. For each value of *N*, 100 simulations were run, initiated at time $t=0$ at the point $\lceil N y_1^*, N y_2^* \rceil$, with a burn-in period of $t_0 = 10$, until extinction or until time $t_{max} = 50000$. Simulations that went extinct before the end of the burn-in period were re-started from $\lceil N y_1^*, N y_2^* \rceil$ at time $t=0$. Maximum likelihood estimates and 95% confidence intervals for $\tau ^{(N)}$ are shown, together with the WKB asymptotic formula (51) (solid line), on a log scale. The WKB formula (51) is seen to provide a good approximation to the simulated mean extinction times, that appears to improve as *N* increases.

## Derivations

We first derive the general results 4 and 5, from which results 1 and 2 follow more or less immediately. Finally, we derive the more precise result 3.

### Derivation of result 4

By van Doorn and Pollett (2013) theorems 6 and 13 and corollary 7, it follows from our assumption 8 that the process ${\varvec{X}}^{(N)} (t)$ is almost surely absorbed at $\textbf{0}$ within finite time, that the elements of the quasistationary distribution ${\varvec{u}}^{(N)}$ satisfy equation (2), and that the time to extinction for the process initiated according to the quasistationary distribution ${\varvec{u}}^{(N)}$ is exponentially distributed with mean $\tau ^{(N)}$ given by equation (3).

Writing ${\varvec{y}}= {\varvec{x}}/ N$, then, following the methodology described in Assaf and Meerson (2017) and references therein, we adopt the WKB *ansatz*. That is, we seek a solution to equation (2) whose components $u_{{\varvec{x}}}^{(N)}$ may be written in the form (4), where, without loss of generality, we set $V ( {\varvec{y}}^* ) = V_0 ( {\varvec{y}}^* ) = 0$, and which is such that $\tau ^{(N)}$ given by equation (3) is sufficiently large for the right hand side of (2) to be neglected. Substituting from (4) into equation (2) and collecting together terms of leading order in *N*, we obtain the Hamilton-Jacobi equation (5). Collecting together second order terms gives the transport equation (6), a first-order linear partial differential equation to be solved for $V_0 ( {\varvec{y}})$, once $V ( {\varvec{y}})$ has been found from equation (5). The derivation thus far, and in particular the form of equations (5), (6), is standard (Assaf and Meerson 2017); the novelty here is in the conditions for analytical, rather than numerical, solution of equations (5), (6), and the form of the analytical solutions, established as follows and in the derivation of result 5 below.

Recalling (assumption 3) that if ${\varvec{l}}\in {{\mathcal {L}}}$ then $- {\varvec{l}}\in {{\mathcal {L}}}$, the Hamilton-Jacobi equation (5) will be satisfied if we can find a function $V ( {\varvec{y}})$ such that $\varvec{\theta }= \frac{\partial V}{\partial {\varvec{y}}}$ satisfies the system of equations (26).

From assumption 3, the system (26) is overdetermined and admits at most one solution $\varvec{\theta }( {\varvec{y}})$ for each ${\varvec{y}}\in {\tilde{S}}^\circ$. Noting that components of ${\varvec{l}}$ are integer-valued for every ${\varvec{l}}\in {{\mathcal {L}}}$, then condition (24) ensures that the system (26) is consistent, so that there exists a unique solution $\varvec{\theta }( {\varvec{y}})$ for each ${\varvec{y}}\in {\tilde{S}}^\circ$.

Next, we require that there exists a scalar potential $V ( {\varvec{y}})$ such that $\frac{\partial V}{\partial {\varvec{y}}} = \varvec{\theta }( {\varvec{y}})$. But for any ${\varvec{l}}_1, {\varvec{l}}_2 \in {{\mathcal {L}}}$, we have from equations (26) that

$$\begin{aligned} {\varvec{l}}_1^T \frac{\partial \varvec{\theta }}{\partial {\varvec{y}}} {\varvec{l}}_2= &  \left( \frac{\partial }{\partial {\varvec{y}}} \ln \left( \frac{\beta _{-{\varvec{l}}_1} ( {\varvec{y}})}{\beta _{{\varvec{l}}_1} ( {\varvec{y}})} \right) \right) ^T {\varvec{l}}_2, \end{aligned}$$

so that applying condition (25),

$$\begin{aligned} {\varvec{l}}_1^T \frac{\partial \varvec{\theta }}{\partial {\varvec{y}}} {\varvec{l}}_2= &  {\varvec{l}}_2^T \frac{\partial \varvec{\theta }}{\partial {\varvec{y}}} {\varvec{l}}_1. \end{aligned}$$

Since ${{\mathcal {L}}}$ spans ${{\mathbb {R}}}^k$, it follows that $\frac{\partial \varvec{\theta }}{\partial {\varvec{y}}}$ is a symmetric matrix, By the Poincaré Lemma (Ciarlet 2013, section 6.17), $\varvec{\theta }( {\varvec{y}})$ is therefore the gradient of a scalar potential $V ( {\varvec{y}})$. The function $V ( {\varvec{y}})$ is given by

52 $$\begin{aligned} V ( {\varvec{y}})= &  \int _{\Gamma } \varvec{\theta }( {\varvec{y}}^\prime ) \cdot d{\varvec{y}}^\prime \text{ for } {\varvec{y}}\in {\tilde{S}}, \end{aligned}$$

where $\Gamma$ is any path from ${\varvec{y}}^*$ to ${\varvec{y}}$ that lies entirely within ${\tilde{S}}$, and the integral is independent of the particular path $\Gamma$.

The function $V ( {\varvec{y}})$ given by equation (52) is not the only solution to equation (5) with $V ( {\varvec{y}}^* ) = 0$; in particular, another solution is given by the function $V ( {\varvec{y}}) = 0$ for all ${\varvec{y}}\in {\tilde{S}}$. To see that equation (52) gives the appropriate solution, we proceed as follows.

Defining constants

$$\begin{aligned} a_{{\varvec{l}}}= &  {\varvec{l}}^T \left. \frac{\partial V}{\partial {\varvec{y}}} \right| _{{\varvec{y}}= {\varvec{y}}^*} \text{ for } {\varvec{l}}\in {{\mathcal {L}}}, \end{aligned}$$

where $V ( {\varvec{y}})$ is given by equation (52), then since ${\varvec{y}}^*$ is an equilibrium point of the system (11) we have

53 $$\begin{aligned} \sum _{{\varvec{l}}\in {{\mathcal {L}}}} \beta _{{\varvec{l}}} ( {\varvec{y}}^* ) a_{{\varvec{l}}} = 0. \end{aligned}$$

Subtracting equation (53) from equation (5) at ${\varvec{y}}^*$, we obtain

54 $$\begin{aligned} \sum _{{\varvec{l}}\in {{\mathcal {L}}}} \beta _{{\varvec{l}}} ( {\varvec{y}}^* ) \left( \exp \left( a_{{\varvec{l}}} \right) - 1 - a_{{\varvec{l}}} \right) = 0. \end{aligned}$$

Now the function $f(a) = e^a - 1 - a$ is non-negative, and equal to zero only when $a=0$, so that the left hand side of equation (54) is a sum of non-negative terms, each term of which must therefore be zero, implying that $a_{{\varvec{l}}} = 0$ for all ${\varvec{l}}\in {{\mathcal {L}}}$. Since ${{\mathcal {L}}}$ spans ${{\mathbb {R}}}^k$, it follows that

55 $$\begin{aligned} \left. \frac{\partial V}{\partial {\varvec{y}}} \right| _{{\varvec{y}}= {\varvec{y}}^*}= &  \textbf{0}, \end{aligned}$$

that is, ${\varvec{y}}^*$ is a stationary point of the function $V ( {\varvec{y}})$ given by equation (52).

Notice also that if ${\tilde{{\varvec{y}}}} \in {\tilde{S}}^\circ$ is any point such that $\varvec{\theta }( {\tilde{{\varvec{y}}}} ) = \left. \frac{\partial V}{\partial {\varvec{y}}} \right| _{{\varvec{y}}= {\tilde{{\varvec{y}}}}} = \textbf{0}$, then from equations (26) it follows that $\beta _{{\varvec{l}}} ( {\tilde{{\varvec{y}}}} ) = \beta _{-{\varvec{l}}} ( {\tilde{{\varvec{y}}}})$ for every ${\varvec{l}}\in \mathcal {L}$ and hence that ${\tilde{{\varvec{y}}}}$ is an equilibrium point of the system (11). But from assumption 7 we know that the system (11) has no equilibrium points within $\tilde{S}^\circ$ other than ${\varvec{y}}^*$, hence the function $V( {\varvec{y}})$ given by equation (52) has no stationary points within $\tilde{S}^\circ$ other than ${\varvec{y}}^*$.

Now with $V ( {\varvec{y}})$ given by equation (52), since $\frac{\partial V}{\partial {\varvec{y}}} = \varvec{\theta }( {\varvec{y}})$, where $\varvec{\theta }( {\varvec{y}})$ is the solution of equations (26), we have

56 $$\begin{aligned} \left. \frac{\partial ^2 V}{\partial {\varvec{y}}^2} \right| _{{\varvec{y}}= {\varvec{y}}^*}= &  \Sigma , \end{aligned}$$

where $\Sigma$ is given by equation (28).

Denote by $J ( {\varvec{y}})$ the Jacobian of the system (11), so that

$$\begin{aligned} J ( {\varvec{y}})= &  \sum _{{\varvec{l}}\in {{\mathcal {L}}}} {\varvec{l}}\left( \frac{\partial \beta _{{\varvec{l}}}}{\partial {\varvec{y}}} \right) ^T, \end{aligned}$$

and define the matrix *G* to be

57 $$\begin{aligned} G= &  \sum _{{\varvec{l}}\in {{\mathcal {L}}}} \beta _{{\varvec{l}}} ( {\varvec{y}}^* ) {\varvec{l}}{\varvec{l}}^T. \end{aligned}$$

Then we have

$$\begin{aligned} G \Sigma= &  \sum _{{\varvec{l}}\in {{\mathcal {L}}}} \beta _{{\varvec{l}}} ( {\varvec{y}}^* ) {\varvec{l}}{\varvec{l}}^T \left. \frac{\partial \varvec{\theta }}{\partial {\varvec{y}}} \right| _{{\varvec{y}}= {\varvec{y}}^*} \\= &  \sum _{{\varvec{l}}\in {{\mathcal {L}}}} \beta _{{\varvec{l}}} ( {\varvec{y}}^* ) {\varvec{l}}\left( \left. \frac{\partial }{\partial {\varvec{y}}} \ln \left( \frac{\beta _{-{\varvec{l}}} ( {\varvec{y}})}{\beta _{{\varvec{l}}} ( {\varvec{y}})} \right) \right| _{{\varvec{y}}= {\varvec{y}}^*} \right) ^T \text{(from } \text{ equations } ~(26)\text{) } \\= &  \sum _{{\varvec{l}}\in {{\mathcal {L}}}} \beta _{{\varvec{l}}} ( {\varvec{y}}^* ) {\varvec{l}}\left( \frac{1}{\beta _{-{\varvec{l}}} ( {\varvec{y}}^* )} \left. \frac{\partial \beta _{-{\varvec{l}}}}{\partial {\varvec{y}}} \right| _{{\varvec{y}}= {\varvec{y}}^*} - \frac{1}{\beta _{{\varvec{l}}} ( {\varvec{y}}^* )} \left. \frac{\partial \beta _{{\varvec{l}}}}{\partial {\varvec{y}}} \right| _{{\varvec{y}}= {\varvec{y}}^*} \right) ^T. \end{aligned}$$

Now from equations (26) and equation (55), we have $\beta _{-{\varvec{l}}} ( {\varvec{y}}^* ) = \beta _{{\varvec{l}}} ( {\varvec{y}}^* )$ for all ${\varvec{l}}\in {{\mathcal {L}}}$, and so

$$\begin{aligned} G \Sigma= &  \sum _{{\varvec{l}}\in {{\mathcal {L}}}} {\varvec{l}}\left( \left. \frac{\partial \beta _{-{\varvec{l}}}}{\partial {\varvec{y}}} \right| _{{\varvec{y}}= {\varvec{y}}^*} - \left. \frac{\partial \beta _{{\varvec{l}}}}{\partial {\varvec{y}}} \right| _{{\varvec{y}}= {\varvec{y}}^*} \right) ^T. \end{aligned}$$

But since ${\varvec{l}}\in {{\mathcal {L}}} \Leftrightarrow - {\varvec{l}}\in {{\mathcal {L}}}$, this can be written as

58 $$\begin{aligned} G \Sigma= &  - \sum _{{\varvec{l}}\in {{\mathcal {L}}}} - {\varvec{l}}\left( \left. \frac{\partial \beta _{-{\varvec{l}}}}{\partial {\varvec{y}}} \right| _{{\varvec{y}}= {\varvec{y}}^*} \right) ^T - \sum _{{\varvec{l}}\in {{\mathcal {L}}}} {\varvec{l}}\left( \left. \frac{\partial \beta _{{\varvec{l}}}}{\partial {\varvec{y}}} \right| _{{\varvec{y}}= {\varvec{y}}^*} \right) ^T \nonumber \\= &  - 2 J ( {\varvec{y}}^* ). \end{aligned}$$

It is clear from the definition (57) that *G* is symmetric and positive-definite, and from the equation (56) that $\Sigma$ is symmetric. From assumption 7 we know that all eigenvalues of $J ( {\varvec{y}}^* )$ have strictly negative real part, so it follows from equation (58) that $\Sigma$ is invertible, and that $\Sigma ^{-1}$ satisfies the Lyapunov equation $J ( {\varvec{y}}^* ) \Sigma ^{-1} + \Sigma ^{-1} J ( {\varvec{y}}^* )^T + G = 0$. From theorems 13.21 and 13.24 of Laub (2005), this Lyapunov equation has a unique solution $\Sigma ^{-1}$, and $\Sigma ^{-1}$ is positive definite.

It follows that ${\varvec{y}}^*$ is a local minimum point of the function $V ( {\varvec{y}})$ given by equation (52), and, since we have shown that this $V( {\varvec{y}})$ has no stationary points within $\tilde{S}^\circ$ other than ${\varvec{y}}^*$, that $V ( {\varvec{y}}) > V ( {\varvec{y}}^* ) = 0$ for all ${\varvec{y}}\in {\tilde{S}} \setminus \{ {\varvec{y}}^* \}$.

To evaluate the constant $M_N$ in the WKB expression (4), again following methodology described in Assaf and Meerson (2017) (although there only for $k=1$ dimensional systems), consider the Taylor series expansion of formula (4) in the vicinity of ${\varvec{y}}^*$. Recalling the conditions $V ( {\varvec{y}}^* ) = V_0 ( {\varvec{y}}^* ) = 0$, and equations (55) and (58), then for $\left| {\varvec{x}}- N {\varvec{y}}^* \right| = O ( \sqrt{N} )$, we obtain

59 $$\begin{aligned} u_{{\varvec{x}}}^{(N)}= &  M_N \exp \left( - \frac{1}{2N} \left( {\varvec{x}}- N {\varvec{y}}^* \right) ^T \Sigma \left( {\varvec{x}}- N {\varvec{y}}^* \right) + o(1) \right) . \end{aligned}$$

Since $\Sigma ^{-1}$ is positive definite, equation (59) represents a multivariate Gaussian distribution with variance matrix $N \Sigma ^{-1}$, normalisation of which implies that

60 $$\begin{aligned} M_N= &  \sqrt{\frac{\text{ det } ( \Sigma )}{(2 \pi N )^k}}. \end{aligned}$$

Notice that the multivariate Gaussian distribution with mean $N {\varvec{y}}^*$ and variance matrix $N \Sigma ^{-1}$ is precisely the usual multivariate normal approximation to the quasistationary distribution ${\varvec{u}}^{(N)}$, which may be obtained as the stationary distribution of an Ornstein-Uhlenbeck process approximating small fluctuations of ${\varvec{X}}^{(N)} (t)$ about $N {\varvec{y}}^*$. Now given the value of $V ( {\varvec{y}})$ in a small neighbourhood of ${\varvec{y}}^*$, the method of characteristics may be used to determine the values of $V ( {\varvec{y}})$ satisfying equation (5) for all ${\varvec{y}}\in {\tilde{S}}$,  (Black and McKane 2011). Hence $V ( {\varvec{y}})$ given by equation (52) is the only solution to equation (5) such that the expression (4) agrees with the usual multivariate normal approximation to ${\varvec{u}}^{(N)}$ as ${\varvec{y}}\rightarrow {\varvec{y}}^*$.

From the leading order term in equation (4) with $V ( {\varvec{y}})$ given by equation (52) we obtain the components of ${\varvec{u}}^{(N)}$ such that equation (2) is asymptotically satisfied to leading order, provided $\tau ^{(N)}$ is sufficiently large that the right hand side of equation (2) may be neglected. Assuming that equation (3) is satisfied here, then from equations (3) and (4), since $\mathcal {L}$ is a finite set, we obtain $\left( \tau ^{(N)} N \right) ^{-1} = \exp \left( - N V( \textbf{0} ) + o (N) \right)$, and since $V ( \textbf{0} ) > 0$, the right hand side of equation (2) is negligible, as required.

We emphasise that the WKB methodology, whilst widely accepted, is not fully rigorous; in particular, note that the pair of equations (2), (3) may not have a unique solution. However, we have exhibited in equation (52) a function $V ( {\varvec{y}})$ such that ${\varvec{u}}^{(N)}$ with components given by equation (4) satisfies equation (2) to leading order in *N* provided the right hand side is negligible, that the right hand side of equation (2) is indeed negligible provided equation (3) holds for this ${\varvec{u}}^{(N)}$, and that this ${\varvec{u}}^{(N)}$ agrees with standard multivariate normal approximation of the quasistationary distribution in the neighbourhood of its mode at $N {\varvec{y}}^*$. It therefore seems reasonable to suppose that ${\varvec{u}}^{(N)}$ with components given by equation (4), where $V ( {\varvec{y}})$ is given by equation (52), does indeed coincide (as the notation implies), to leading order, with the limiting conditional distribution defined in assumption 8. The relationship (27) follows, provided the integral converges (noting that the integrand is undefined at the origin).

### Derivation of result 5

Now consider the function $V_0 ( {\varvec{y}})$. Since $\varvec{\theta }( {\varvec{y}}) = \frac{\partial V}{\partial {\varvec{y}}}$ satisfies equations (26), the transport equation (6) simplifies to

61 $$\begin{aligned} \sum _{{\varvec{l}}\in {{\mathcal {L}}}} \beta _{-{\varvec{l}}} ( {\varvec{y}}) {\varvec{l}}^T \left( \frac{\partial V_0}{\partial {\varvec{y}}} - \frac{1}{2} \frac{\partial }{\partial {\varvec{y}}} \ln \left( \beta _{-{\varvec{l}}} ( {\varvec{y}}) \beta _{{\varvec{l}}} ( {\varvec{y}}) \right) \right)= &  0. \end{aligned}$$

Equation (61) will be satisfied if we can find a function $V_0 ( {\varvec{y}})$ such that $\varvec{\theta }^{0} = \frac{\partial V_0}{\partial {\varvec{y}}}$ satisfies the system of equations (31). Arguing as in the derivation of result 4, condition (29) ensures that the linear system (31) admits a unique solution $\varvec{\theta }^{0} ( {\varvec{y}})$, and condition (30) ensures, via the Poincaré Lemma, that $\varvec{\theta }^{0} ( {\varvec{y}})$ is the gradient of a scalar potential $V_0 ( {\varvec{y}})$, with

62 $$\begin{aligned} V_0 ( {\varvec{y}})= &  \int _{\Gamma ^\prime } \varvec{\theta }^{0} ( {\varvec{y}}^\prime ) \cdot d {\varvec{y}}^\prime \text{ for } {\varvec{y}}\in {\tilde{S}}^\circ , \end{aligned}$$

where $\Gamma ^\prime$ is any path from ${\varvec{y}}^*$ to ${\varvec{y}}$ that lies entirely within ${\tilde{S}}$, and the integral is independent of the particular path $\Gamma ^\prime$.

Since ${{\mathcal {L}}}$ spans ${{\mathbb {R}}}^k$, any ${\varvec{y}}\in {\tilde{S}}$ may be written in the form ${\varvec{y}}= {\varvec{y}}^* + \sum _{i=1}^n a_i {\varvec{l}}_i$ for some (not necessarily unique) $a_1, a_2, \ldots , a_n \in {{\mathbb {R}}}$ and ${\varvec{l}}_1, {\varvec{l}}_2, \ldots , {\varvec{l}}_n \in {{\mathcal {L}}}$ such that ${\varvec{y}}^* + \sum _{i=1}^j a_i {\varvec{l}}_i \in {\tilde{S}}$ for $j=1,2,\ldots ,n$. From equations (31), we have

$$\begin{aligned} {\varvec{l}}^T \frac{\partial }{\partial {\varvec{y}}} \left( V_0 - \frac{1}{2} \ln \left( \beta _{-{\varvec{l}}} ( {\varvec{y}}) \beta _{{\varvec{l}}} ( {\varvec{y}}) \right) \right)= &  0 \text{ for } \text{ all } {\varvec{l}}\in {{\mathcal {L}}}. \end{aligned}$$

Starting from ${\varvec{y}}^*$ and integrating along each direction ${\varvec{l}}_1, {\varvec{l}}_2, \ldots , {\varvec{l}}_n$ in turn, we thus obtain

63 $$\begin{aligned} V_0 ( {\varvec{y}})= &  \frac{1}{2} \sum _{j=1}^n \ln \left( \frac{\beta _{-{\varvec{l}}_j} \left( {\varvec{y}}^* + \sum _{i=1}^j a_i {\varvec{l}}_i \right) \beta _{{\varvec{l}}_j} \left( {\varvec{y}}^* + \sum _{i=1}^j a_i {\varvec{l}}_i \right) }{\beta _{-{\varvec{l}}_j} \left( {\varvec{y}}^* + \sum _{i=1}^{j-1} a_i {\varvec{l}}_i \right) \beta _{{\varvec{l}}_j} \left( {\varvec{y}}^* + \sum _{i=1}^{j-1} a_i {\varvec{l}}_i \right) } \right) . \end{aligned}$$

Since the integral in equation (62) is independent of the particular path chosen, the expression (63) is correspondingly independent of the particular representation ${\varvec{y}}= {\varvec{y}}^* + \sum _{i=1}^n a_i {\varvec{l}}_i$.

Notice that the expression (63) diverges for ${\varvec{y}}\in \partial {\tilde{S}}$. For $d ( {\varvec{y}}, \partial {\tilde{S}} ) \ne o(1)$, substituting for $V ( {\varvec{y}})$, $V_0 ( {\varvec{y}})$ and $M_N$ from equations (52), (63) and (60) into the WKB formula (4), the result follows (while noting the same ‘semi-rigorous’ nature of the WKB methodology as in the derivation of result 4).

### Derivation of result 1

In the case ${{\mathcal {L}}} = \{ {\varvec{e}}_i, - {\varvec{e}}_i: i=1,2,\ldots ,k \}$, condition (24) is automatically satisfied, and condition (25) reduces to condition (15). The system of equations (26) reduces to $\theta _i ( {\varvec{y}}) = h_i ( {\varvec{y}})$ for $i=1,2,\ldots ,k$, where $h_i ( {\varvec{y}})$ is given by equations (14), and so the function $V ( {\varvec{y}})$ in the derivation of result 4 is given by

64 $$\begin{aligned} V ( {\varvec{y}})= &  \int _{\Gamma } {\varvec{h}}( {\varvec{y}}^\prime ) \cdot d{\varvec{y}}^\prime \text{ for } {\varvec{y}}\in {\tilde{S}} \end{aligned}$$

where $\Gamma$ is any path from ${\varvec{y}}^*$ to ${\varvec{y}}$ that lies entirely within ${\tilde{S}}$, and the integral is independent of the particular path $\Gamma$.

The integrand in equation (64) is undefined at the origin. By assumptions 4 and 5, recalling the definitions (12), we have that $b_{ij} \ge 0$, $d_i \ge 0$ for all *i*, *j*, and that $\left. \frac{\partial \beta _{-{\varvec{e}}_i}}{\partial y_j} \right| _{{\varvec{y}}= \textbf{0}} = 0$ for $i \ne j$. We now make the further supposition, condition (13), that $d_i > 0$ for all *i*, and that for each *i*, there exists some *j* such that $b_{ij} > 0$. Consider approach to the origin along the path ${\varvec{y}}= {\hat{y}} \varvec{\xi }$ for some fixed $\varvec{\xi }= \left( \xi _1 , \xi _2 , \ldots , \xi _k \right)$ with $\xi _i > 0$ for all *i* and $\sum _{i=1}^k \xi _i = 1$. Applying l’Hôpital’s rule along this path, we have

$$\begin{aligned} \lim _{{\hat{y}} \rightarrow 0} \frac{\beta _{-{\varvec{e}}_i} ( {\hat{y}} \varvec{\xi })}{\beta _{{\varvec{e}}_i} ( {\hat{y}} \varvec{\xi })}= &  \frac{\xi _i d_i}{\sum _{j=1}^k \xi _j b_{ij}}, \end{aligned}$$

a finite and non-zero limit. Hence along this path, for each *i*, $h_i ( {\varvec{y}})$ defined by (14) converges to a finite limit, and hence for ${\varvec{y}}= \textbf{0}$, the integral in equation (64) converges.

Result 1 now follows from result 4.

### Derivation of result 2

In the case ${{\mathcal {L}}} = \{ {\varvec{e}}_i, - {\varvec{e}}_i: i=1,2,\ldots ,k \}$, condition (29) is automatically satisfied, and condition (30) reduces to condition (19). The system of equations (31) reduces to $\theta _i^{0} ( {\varvec{y}}) = h_i^0 ( {\varvec{y}})$ for $i=1,2,\ldots ,k$, where $h_i^0 ( {\varvec{y}})$ is given by equations (18), and result 2 thus follows from result 5.

### Derivation of result 3

To approximate the mean extinction time $\tau ^{(N)}$ using formula (3), we need to be able to approximate the quasistationary probabilities $u_{{\varvec{e}}_i}^{(N)}$ for $i=1,2,\ldots ,k$. Since $V_0 ( {\varvec{y}})$ diverges for ${\varvec{y}}\in \partial {\tilde{S}}$, we cannot directly make use of the WKB approximation (20). Instead, we will now seek an approximation for $u_{{\varvec{x}}}^{(N)}$ valid for $| {\varvec{x}}| = O(1)$. In order to normalise this approximation, we will make use of the WKB approximation (20). The methodology that we follow has been previously applied to $k=1$ dimensional models, see Assaf and Meerson (2010, 2017) and references therein, and to one specific multidimensional model (our example 1) in Clancy (2018b); we extend the approach to cover a more general class of models in $k \ge 1$ dimensions.

With the convention that $u_{{\varvec{x}}}^{(N)} = 0$ for ${\varvec{x}}\notin C^{(N)}$, the exact equation (2) may be written as

65 $$\begin{aligned} \sum _{i=1}^k \left( u_{{\varvec{x}}- {\varvec{e}}_i}^{(N)} \beta _{{\varvec{e}}_i} \left( \frac{{\varvec{x}}- {\varvec{e}}_i}{N} \right) + u_{{\varvec{x}}+ {\varvec{e}}_i}^{(N)} \beta _{-{\varvec{e}}_i} \left( \frac{{\varvec{x}}+ {\varvec{e}}_i}{N} \right) - u_{{\varvec{x}}}^{(N)} \left( \beta _{{\varvec{e}}_i} \left( \frac{{\varvec{x}}}{N} \right) + \beta _{-{\varvec{e}}_i} \left( \frac{{\varvec{x}}}{N} \right) \right) \right) \nonumber \\ &  \hspace{-65mm} = \;\; - ( \tau ^{(N)} N )^{-1} u_{{\varvec{x}}}^{(N)} \text{ for } {\varvec{x}}\in C^{(N)}. \end{aligned}$$

Supposing, as before, that $\tau ^{(N)}$ is sufficiently large for the right-hand side of equation (65) to be neglected, taking the linear approximation to the left-hand side, recalling the definitions (12) of the constants $b_{ij}, d_i$, and writing $\varvec{b}^{(i)} = \left( b_{i1}, b_{i2}, \ldots , b_{ik} \right)$ for $i=1,2,\ldots ,k$, we obtain the asymptotic balance equation

66 $$\begin{aligned} \sum _{i=1}^k \left( {\tilde{u}}_{{\varvec{x}}- {\varvec{e}}_i} ( {\varvec{x}}- {\varvec{e}}_i )^T \varvec{b}^{(i)} + {\tilde{u}}_{{\varvec{x}}+ {\varvec{e}}_i} ( x_i + 1 ) d_i \right) - {\tilde{u}}_{{\varvec{x}}} \sum _{i=1}^k \left( {\varvec{x}}^T \varvec{b}^{(i)} + x_i d_i \right)= &  0,\nonumber \\ \end{aligned}$$

where $\{ {\tilde{u}}_{{\varvec{x}}}: {\varvec{x}}\in {{\mathbb {Z}}}_+^k \}$ denotes the solution of the linearised balance equation.

Recalling our assumptions that $b_{ii}, d_i > 0$ for $i=1,2,\ldots ,k$, then under the condition (21), which ensures that the linear approximating process satisfies the Kolmogorov criterion (9), the asymptotic balance equation (66) has solution

67 $$\begin{aligned} {\tilde{u}}_{{\varvec{x}}}= &  \Lambda \, \frac{1}{\sum _{i=1}^k x_i} \frac{\left( \sum _{i=1}^k x_i \right) !}{\prod _{i=1}^k x_i !} \prod _{i=1}^k b_{ii}^{x_i} \left( \prod _{i=1}^k \left( d_i \right) ^{-x_i} - \prod _{i=1}^k \left( D + d_i \right) ^{-x_i} \right) \end{aligned}$$

where $\Lambda$ is a normalising constant to be found, and *D* satisfies equation (22). The first component of the solution (67) is found by solving the detailed balance equations (10) corresponding to equation (66), and the other component by analogy with the solution for the case $k = 1$ given in Assaf and Meerson (2010), see also Clancy (2018b).

Local instability of the equilibrium point at ${\varvec{y}}= \textbf{0}$ for the system (11) (assumption 7) implies that at least one eigenvalue of the Jacobian of system (11) at the origin, $J ( \textbf{0} )$, has positive real part. Now $J ( { \textbf{0}} )$ has components

$$\begin{aligned} J_{ij} ( \textbf{0} )= &  b_{ii} - d_i \delta _{ij} \text{ for } i,j = 1,2,\ldots ,k, \end{aligned}$$

where $\delta _{ij}$ is the Kronecker delta. It follows from theorem A.1 of Diekmann et al. (2010) that $\sum _{i=1}^k \left( b_{ii} / d_i \right) > 1$, and hence that equation (22) has a unique positive solution *D*.

To evaluate the normalising constant $\Lambda$ in formula (67), we match the approximation (67) with our WKB approximation (20) in their common range of validity. First, consider the approximation (67) for large $| {\varvec{x}}|$. Applying Stirling’s formula to the factorial terms, and recalling that $D>0$, we obtain

$$\begin{aligned} {\tilde{u}}_{{\varvec{x}}}\sim &  \Lambda \, \left( \sum _{i=1}^k x_i \right) ^{\sum _{i=1}^k x_i} \sqrt{\frac{1}{(2 \pi )^{k-1} \left( \sum _{i=1}^k x_i \right) \prod _{i=1}^k x_i}} \, \prod _{i=1}^k \left( \frac{b_{ii}}{x_i d_i} \right) ^{x_i}. \end{aligned}$$

Along the specific trajectory ${\varvec{x}}= {\hat{x}} \varvec{\xi }$, where $\varvec{\xi }= \left( \xi _1, \xi _2, \ldots , \xi _k \right)$ is fixed, with $\xi _1, \xi _2, \ldots , \xi _k > 0$ and $\sum _{i=1}^k \xi _i = 1$, then as ${\hat{x}} \rightarrow \infty$ we have

68 $$\begin{aligned} {\tilde{u}}_{{\varvec{x}}}\sim &  \Lambda \, \sqrt{\frac{1}{(2 \pi )^{k-1} {\hat{x}}^{k+1} \prod _{i=1}^k \xi _i}} \, \prod _{i=1}^k \left( \frac{b_{ii}}{\xi _i d_i} \right) ^{{\hat{x}} \xi _i}. \end{aligned}$$

Returning to the WKB approximation (20), consider the trajectory ${\varvec{y}}= {\hat{y}} \varvec{\xi }$ as ${\hat{y}} \downarrow 0$, where ${\varvec{y}}= {\varvec{x}}/ N$. Under the assumption (21), applying l’Hôpital’s rule along the trajectory ${\varvec{y}}= {\hat{y}} \varvec{\xi }$ to the derivatives $\partial V / \partial y_i = h_i ( {\varvec{y}})$ given by (14), we have

$$\begin{aligned} \frac{\partial V}{\partial y_i}= &  \ln \left( \frac{\beta _{-{\varvec{e}}_i} ( {\hat{y}} \varvec{\xi })}{\beta _{{\varvec{e}}_i} ( {\hat{y}} \varvec{\xi })} \right) \rightarrow \ln \left( \frac{\xi _i d_i}{b_{ii}} \right) \text{ as } {\hat{y}} \downarrow 0, \end{aligned}$$

so that Taylor series expansion of $V ( {\varvec{y}})$ in the range $| {\varvec{x}}| = O ( \sqrt{N} )$ gives

69 $$\begin{aligned} \exp ( - N V ( {\varvec{y}}) )\sim &  \exp \left( - N V ( \textbf{0} ) - {\hat{x}} \sum _{i=1}^k \xi _i \ln \left( \frac{\xi _i d_i}{b_{ii}} \right) + O (1/N) \right) \nonumber \\= &  \prod _{i=1}^k \left( \frac{b_{ii}}{\xi _i d_i} \right) ^{{\hat{x}} \xi _i} \exp \left( - N V ( \textbf{0} ) + O (1/N) \right) . \end{aligned}$$

Under the assumption (21), we also have that for $i=1,2,\ldots ,k$,

70 $$\begin{aligned} \beta _{-{\varvec{e}}_i} ( y_1, \ldots , y_i, y_{i+1}^*, \ldots , y_k^* )= &  {\hat{y}} \xi _i \left. \frac{\partial \beta _{-{\varvec{e}}_i}}{\partial y_i} \right| _{{\varvec{y}}= (0,\ldots ,0,y_{i+1}^*, \ldots , y_k^* )} + O ( {\hat{y}}^2 ), \end{aligned}$$

and

71 $$\begin{aligned} \beta _{{\varvec{e}}_k} ( {{\varvec{y}}} )= &  b_{kk} {\hat{y}} + O ( {\hat{y}}^2 ). \end{aligned}$$

Substituting from equations (69, 70, 71) into the WKB approximation formula (20) yields, along the trajectory ${\varvec{x}}= {\hat{x}} \varvec{\xi }$ in the range $| {\varvec{x}}| = O ( \sqrt{N} )$,

72 $$\begin{aligned} {\tilde{u}}_{{\varvec{x}}}\sim &  \sqrt{\frac{N \text{ det } ( \Sigma )}{(2 \pi )^k {\hat{x}}^{k+1}} {\prod _{i=1}^k \beta _{{\varvec{e}}_i} \left( 0, \ldots , 0, y_i^*, \ldots , y_k^* \right) \beta _{-{\varvec{e}}_i} \left( 0, \ldots , 0, y_i^*, \ldots , y_k^* \right) \over b_{kk} \prod _{i=1}^k \left( \xi _i \left. \frac{\partial \beta _{-{\varvec{e}}_i}}{\partial y_i} \right| _{{\varvec{y}}= (0, \ldots , 0, y^*_{i+1}, \ldots , y^*_k )} \right) \prod _{i=1}^{k-1} \beta _{{\varvec{e}}_i} \left( 0, \ldots , 0, y_{i+1}^*, \ldots , y_k^* \right) }} \nonumber \\  &  {} \times \prod _{i=1}^k \left( \frac{b_{ii}}{\xi _i d_i} \right) ^{{\hat{x}} \xi _i} \exp \left( - N V ( \textbf{0} ) \right) . \end{aligned}$$

Matching the expressions (68) and (72), we see that terms in ${\hat{x}}$ and $( \xi _1, \xi _2, \ldots , \xi _k )$ do indeed match, and we obtain the normalising constant $\Lambda$ as

73 $$\begin{aligned} \Lambda= &  \sqrt{\frac{N \text{ det } ( \Sigma )}{2 \pi } {\prod _{i=1}^k \beta _{{\varvec{e}}_i} \left( 0, \ldots , 0, y_i^*, \ldots , y_k^* \right) \beta _{-{\varvec{e}}_i} \left( 0, \ldots , 0, y_i^*, \ldots , y_k^* \right) \over b_{kk} \prod _{i=1}^k \left. \frac{\partial \beta _{-{\varvec{e}}_i}}{\partial y_i} \right| _{{\varvec{y}}= (0, \ldots , 0, y^*_{i+1}, \ldots , y^*_k )} \prod _{i=1}^{k-1} \beta _{{\varvec{e}}_i} \left( 0, \ldots , 0, y_{i+1}^*, \ldots , y_k^* \right) }} \nonumber \\ &  {} \times \exp \left( - N V( \textbf{0} ) \right) . \end{aligned}$$

Now substituting for ${\tilde{u}}_{{\varvec{e}}_i}$ from equation (67) into equation (3), noting that $\beta _{-{\varvec{e}}_i} \left( \frac{{\varvec{e}}_i}{N} \right) = d_i + O(1/N)$, and recalling the relationship (22), we obtain

74 $$\begin{aligned} \tau ^{(N)}= &  \left( N \sum _{i=1}^k u_{{\varvec{e}}_i}^{(N)} \beta _{-{\varvec{e}}_i} \left( \frac{{\varvec{e}}_i}{N} \right) \right) ^{-1} \nonumber \\\sim &  \left( \Lambda \sum _{i=1}^k b_{ii} \left( 1 - \frac{d_i}{D + d_i} \right) \right) ^{-1} \nonumber \\= &  \left( \Lambda D \right) ^{-1}. \end{aligned}$$

From equations (74), (73) and  (64), the result (23) follows.

#### Remark 6

We see in the derivations of results 4 and 5, recalling remark 5, that in this context, the well known result from vector calculus (the Poincaré Lemma) that “a vector field (here $\varvec{\theta }( {\varvec{y}})$, $\varvec{\theta }^0 ( {\varvec{y}})$, respectively) on a simply connected open subset of ${{\mathbb {R}}}^k$ is conservative (that is, there exists a scalar potential $V ( {\varvec{y}})$, $V_0 ( {\varvec{y}})$, respectively, such that $\varvec{\theta }( {\varvec{y}}) = \partial V / \partial {\varvec{y}}$, $\varvec{\theta }^0 ( {\varvec{y}}) = \partial V_0 / \partial {\varvec{y}}$) if and only if it is irrotational (equivalent to condition (25), (30), respectively)” asymptotically parallels the well known result in Markov process theory that “the stationary distribution of an irreducible, positive recurrent Markov process satisfies the detailed balance equations (10) if and only if the transition rates of the process satisfy the Kolmogorov criterion (9).” Since we are concerned with quasistationary distributions rather than stationary distributions, and our results are asymptotic rather than exact, we require that the set of states $C^{(N)} = S^{(N)} \!\setminus \! \{ 0 \}$ be a communicating class, rather than that the process ${\varvec{X}}^{(N)} (t)$ be irreducible.

## Data Availability

Not applicable

## References

[CR1] Andersson H, Britton T (2000) Stochastic epidemics in dynamic populations: quasi-stationarity and extinction. J Math Biol 41:559–580 11196585 10.1007/s002850000060

[CR2] Assaf M, Meerson B (2010) Extinction of metastable stochastic populations. Phys Rev E 81:021116 10.1103/PhysRevE.81.021116 20365539

[CR3] Assaf M, Meerson B (2017) WKB theory of large deviations in stochastic populations. J Phys A: Math Theor 50:263001

[CR4] Ball FG, Clancy D (2023) Asymptotic persistence time formulae for multitype birth-death processes. J Appl Probab 60:895–920

[CR5] Bauver M, Forgoston E, Billings L (2016) Computing the optimal path in stochastic dynamical systems. Chaos 26:083101 27586597 10.1063/1.4958926

[CR6] Berglund N (2013) Kramers’ law: validity, derivations and generalisations. Markov Process Related Fields 19:459–490

[CR7] Black AJ, McKane AJ (2011) WKB calculation of an epidemic outbreak distribution. J Statist Mech 12:P12006

[CR8] Champagnat N, Villemonais D (2023) General criteria for the study of quasi-stationarity. Electron J Probab 28:1–84

[CR9] Chazottes JB, Collet P, Méléard S (2019) On time scales and quasi-stationary distributions for multitype birth-and-death processes. Ann Inst H Poincaré Probab Statist 55:2249–2294

[CR10] Ciarlet PG (2013) Linear and Nonlinear Functional Analysis with Applications. SIAM, Philadelphia

[CR11] Clancy D (2018a) Persistence time of SIS infections in heterogeneous populations and networks. J Math Biol 77:545–570 10.1007/s00285-018-1222-1 PMC6132977 29476196

[CR12] Clancy D (2018b) Precise estimates of persistence time for SIS infections in heterogeneous populations. Bull Math Biol 80:2871–2896 10.1007/s11538-018-0491-6 30206808

[CR13] Clancy D, Stewart JJH (2024) Extinction in host-vector infection models and the role of heterogeneity. Math Biosci 367:109108 38070764 10.1016/j.mbs.2023.109108

[CR14] Clancy D, Stewart JJH (2025) Computing the extinction path for epidemic models. Math Biosci 386:109454 40348331 10.1016/j.mbs.2025.109454

[CR15] Clancy D, Tjia E (2018) Approximating time to extinction for endemic infection models. Methodol Comput Appl Probab 20:1043–1067

[CR16] Darroch JN, Seneta E (1967) On quasi-stationary distributions in absorbing continuous-time finite Markov chains. J Appl Probab 4:192–196

[CR17] Diekmann O, Heesterbeek JAP, Roberts MG (2010) The construction of next-generation matrices for compartmental epidemic models. J R Soc Interface 7:873–885 19892718 10.1098/rsif.2009.0386 PMC2871801

[CR18] Doering CR, Sargasyan KV, Sander LM (2005) Extinction times for birth-death processes: exact results, continuum asymptotics, and the failure of the Fokker-Planck approximation. Multiscale Model Simul 3:283–299

[CR19] van Doorn EA, Pollett PK (2013) Quasi-stationary distributions for discrete-state models. Eur J Oper Res 230:1–14

[CR20] Dykman MI, Mori E, Ross J, Hunt PM (1994) Large fluctuations and optimal paths in chemical kinetics. J Chem Phys 100:5735–5750

[CR21] Ethier SN, Kurtz TG (2005) Markov Processes: Characterization and Convergence. Wiley, New York

[CR22] Iglehart DL (1964) Reversible competition processes. Z Wahrseheinliehkeitstheorie 2:314–331

[CR23] Jia C, Jiang DQ, Li Y (2021) Detailed balance, local detailed balance, and global potential for stochastic chemical reaction networks. Adv Appl Probab 53:886–922

[CR24] Kamenev A, Meerson B (2008) Extinction of an infectious disease: a large fluctuation in a nonequilibrium system. Phys Rev E 77:061107 10.1103/PhysRevE.77.061107 18643217

[CR25] Kelly FP (2011) Reversibility and Stochastic Networks. Cambridge University Press, Cambridge

[CR26] Lajmanovich A, Yorke JA (1976) A deterministic model for gonorrhea in a nonhomogeneous population. Math Biosci 28:221–236

[CR27] Laub AJ (2005) Matrix Analysis for Scientists and Engineers. SIAM publications, Philadelphia

[CR28] Lloyd AL, Zhang J, Morgan Root A (2007) Stochasticity and heterogeneity in host-vector models. J Roy Soc Interface 4:851–863 17580290 10.1098/rsif.2007.1064 PMC2394551

[CR29] Méléard S, Villemonais D (2012) Quasi-stationary distributions and population processes. Probab Surv 9:340–410

[CR30] Nåsell I (1991) On the quasistationary distribution of the Ross malaria model. Math Biosci 107:187–208 1806113 10.1016/0025-5564(91)90004-3

[CR31] Nåsell I (1999) On the time to extinction in recurrent epidemics. J Roy Statistic Soc B 61:309–330

[CR32] Nåsell I (2011) Extinction and Quasi-stationarity in the Stochastic Logistic SIS Model. Springer, Berlin/Heidelberg

[CR33] Nieddu GT, Billings L, Kaufman JH, Forgoston E, Bianco S (2017) Extinction pathways and outbreak vulnerability in a stochastic Ebola model. J Roy Soc Interface 14:20160847 10.1098/rsif.2016.0847 PMC5332568 28202592

[CR34] Ovaskainen O, Meerson B (2010) Stochastic models of population extinction. Trends in Ecology & Evolution 25:643–652 20810188 10.1016/j.tree.2010.07.009

[CR35] Reuter GEH (1961) Competition processes. Proc 4th Berkley Symp Math Statist Prob 2:421–430

[CR36] Ross JV (2006) A stochastic metapopulation model accounting for habitat dynamics. J Math Biol 52:788–806 16521025 10.1007/s00285-006-0372-8

[CR37] Stirk ER, Lythe G, van den Berg HA, Hurst GAD, Molina-París C (2010) The limiting conditional probability distribution in a stochastic model of T cell repertoire maintenance. Math Biosci 224:74–86 10.1016/j.mbs.2009.12.004 20060005

[CR38] Sundberg R (2001) Comparison of confidence procedures for type I censored exponential lifetimes. Lifetime Data Anal 7:393–413 11763546 10.1023/a:1012500932414

[CR39] Vadillo F (2021) On deterministic and stochastic multiple pathogen epidemic models. Epidemiologia 2:325–337 36417229 10.3390/epidemiologia2030025 PMC9620904

[CR40] Wang X, Gautam R, Pinedo PJ, Allen LJS, Ivanek R (2014) A stochastic model for transmission, extinction and outbreak of Escherichia coli O157:H7 in cattle as affected by ambient temperature and cleaning practices. J Math Biol 69:501–532 10.1007/s00285-013-0707-1 23864122

